# A Spatial Control for Correct Timing of Gene Expression during the *Escherichia coli* Cell Cycle

**DOI:** 10.3390/genes8010001

**Published:** 2016-12-23

**Authors:** Yuan Yao, Lifei Fan, Yixin Shi, Ingvild Odsbu, Morigen

**Affiliations:** 1 School of Life Sciences, Inner Mongolia University, Hohhot 010021, China; zhjyq129@163.com (Y.Y.); lifei.fan@life.imu.edu.cn (L.F.); yixin.shi@asu.edu (Y.S.); 2 School of Life Sciences, Arizona State University, Tempe, AZ 85287-4501, USA; 3 Department of Public Health Sciences, Karolinska Institutet, SE-171 77 Stockholm, Sweden; Ingvild.odsbu@gmail.com

**Keywords:** cell-cycle-dependent, co-localization, TorR focus, nucleoid, spatial control, gene expression

## Abstract

Temporal transcriptions of genes are achieved by different mechanisms such as dynamic interaction of activator and repressor proteins with promoters, and accumulation and/or degradation of key regulators as a function of cell cycle. We find that the TorR protein localizes to the old poles of the *Escherichia coli* cells, forming a functional focus. The TorR focus co-localizes with the nucleoid in a cell-cycle-dependent manner, and consequently regulates transcription of a number of genes. Formation of one TorR focus at the old poles of cells requires interaction with the MreB and DnaK proteins, and ATP, suggesting that TorR delivery requires cytoskeleton organization and ATP. Further, absence of the protein–protein interactions and ATP leads to loss in function of TorR as a transcription factor. We propose a mechanism for timing of cell-cycle-dependent gene transcription, where a transcription factor interacts with its target genes during a specific period of the cell cycle by limiting its own spatial distribution.

## 1. Introduction

A complex regulatory network coordinates the progression of the cell cycle by temporal and spatial cell-cycle-dependent transcription both in bacteria and yeast [1,2]. In *Caulobacter crescentus*, the expression of 15%–19% of the genes varies with the cell cycle [3,4], suggesting a strong correlation between specific gene transcription and the progression of the cell cycle. A key cell cycle regulator, the response regulator CtrA of the CckA/CtrA two-component signal transduction system, controls the expression of a number of genes in a cell-cycle-dependent manner [4,5]. The CtrA protein is degraded in G1 phase, derepressing expression of the *dnaA* gene (encoding the initiator for replication) and the *gcrA* gene (encoding another regulator, the GcrA protein). After initiation of chromosome replication, triggered by DnaA, *CtrA* is transcribed by stimulation of GcrA, suppressing another round of replication [6]. In *Escherichia coli*, an oscillatory pattern in transcription of *DnaA* [7], *gidA*, *mioC* [8] and the *ftsQAZ* operon [9,10] is correlated with replication initiation and cell division. The *E. coli* DnaA can be found in two forms: ATP-DnaA and ADP-DnaA. The ATP-DnaA is active for initiation of replication and is a strong suppressor of several genes [11]. It is hydrolysed to an ADP-bound form during chromosome replication by the RIDA (regulatory inactivation of DnaA) process, and the activity of RIDA depends on cell-cycle progression [12]. Thus, DnaA regulates transcription of a number of genes in a cell-cycle-dependent manner. It has been found that: (i) a dynamic interaction of activator and repressor proteins with promoters; (ii) accumulation and/or degradation of key regulators; and (iii) a dynamic change in protein activity within the cell cycle, control the expression of key genes in a cell-cycle-dependent manner.

Bacterial polarization is determined by dynamic changes in the subcellular localization of the signal transduction and cytoskeleton proteins as well as specific regions of the chromosome [13]. Indeed, the histidine kinase CckA from the CckA/CtrA two-component signal transduction system is transiently localized to the cell pole at the time of CtrA synthesis to activate CtrA by phosphorylation. Thus, signaling from proteins (PleC, DivJ, CckA, CtrA and DivK) arrested at the cell pole coupled to cell-cycle-controlled activation or proteolysis regulates cell cycle progression and polarization in *C. crescentus* [13]. Similarly, the *E. coli* CheA/CheY and CheA/CheB two-component signal transduction systems decide the cellular chemotaxis by polar localization [14]. The dynamic changes in subcellular localization of MinCDE determine the site of FtsZ-ring formation, ensuring that cell division occurs at the middle of the cell [15,16].

In *E. coli*, the TorS/TorR two-component signal transduction proteins and periplasmic TorT protein make up the trimethylamine oxide (TMAO)-regulatory system which regulates the expression of the *torCAD* operon encoding the TMAO-reductase anaerobic respiratory system [17]. TorS belongs to an unorthodox sensor (histidine kinase) and its cognate response regulator is TorR. TorS senses TMAO and transphosphorylates the TorR, the latter, in turn, regulates transcription of the *tor* operon [18]. Compared with the TMAO effect on gene regulation, anaerobic control is weak for the *tor* operon [18]. Surprisingly, it has also been observed that unphosphorylated TorR binds to the high affinity TorR-binding sites in the *tor* promoter region as does phosphorylated TorR, preventing RNA polymerase from binding to the promoter in any growth conditions [18].

In a screening for candidates that can be involved in control of initiation of chromosomal replication, we found that absence of TorR protein led to an early initiation of replication and the effect was indirect [19]. Interestingly, the fluorescence microscopy analysis showed that the TorR-green fluorescent protein (GFP) fusion protein produced from a plasmid under control of the *lac* promoter [20] localized at the old pole of the cells as a focus. Further, we found that TorR co-localized with the nucleoid in a cell-cycle-dependent manner, and the co-localization in turn regulated transcription of a number of genes. The MreB and DnaK proteins were found to interact with TorR and were required for formation of one TorR focus at the old poles of cells and its regulatory function on gene expression. Therefore, we propose a mechanism for correct timing of gene expression during the cell cycle, where a transcription factor interacts with its target genes during a specific period of cell cycle by limiting its own spatial distribution.

## 2. Materials and Methods

### 2.1. Bacterial Strains and Plasmids

Bacterial strains which are *E. coli* K-12 and plasmids used in this study, are listed in Tables S3 and S4, respectively. Primers used are in Table S5. The *torR:: kan^R^* allele was P1 transduced into *dnaC2*, *ftsZ84* and *minCDE* mutants, respectively [21]. In order to express IbpA-mCherry fusion protein, the *ibpA* gene with its native promoter region was generated by PCR using chromosomal DNA as a template and a pair of primers of ibpA-for and ibpA-rev. The *mCherry* gene was PCR amplified from plasmid pcDNA3-mCherry with the mix2-for and mix2-rev primers. Then, the *ibpA* gene with its promoter was fused to the N-terminus of the *mCherry* gene through a HindIII site, and the resultant *ibpA*-mCherry fragment was inserted into pACYC177 at XhoI and BamHI sites, constructing a plasmid, pIbpA-mCherry. For expression of the TorR-mCherry fusion protein, the *torR* gene with its native promoter was PCR amplified using chromosomal DNA as a template and a pair of primers of torR1-for and torR1-rev. The *mCherry* gene was obtained as described above. Subsequently, the *torR* gene with its promoter was fused to the N-terminus of *mCherry* gene through a KpnI site, and the resultant *torR*-mCherry fragment was inserted into pACYC177 at HindIII and BamHI sites, resulting in plasmid pTorR-mCherry. For expression of the MreB-mCherry fusion protein, the *mreB* gene with its native promoter was PCR amplified using chromosomal DNA as template and a pair of primers of mreB-for and mreB -rev. Subsequently, the *mreB* gene with its promoter was fused to the N-terminus of *mCherry* gene through a HindIII site, and the resultant *mreB*-mCherry fragment was inserted into pACYC177 at XhoI and BamHI sites, resulting in plasmid pMreB-mCherry. For the bacterial two-hybrid (BACTH) [22,23] studies, the T25 or T18 domain was, respectively, fused to the N- or C-terminus of the target gene (*torR*, *mreB*, *dnaK* or *ftsZ*) through a proper pair of restriction enzymes as described in Table S5. The *torR* gene was amplified as described above and inserted into the pKNT25 at HindIII and BamHI sites, fusing the C-terminus of TorR to the N-terminus of the T25 domain. In the other constructions, *mreB*, *dnaK* or the *ftsZ* genes were PCR amplified using the primers mre-for and mre-rev for *mreB*, dnaK-for and dnaK-rev for *dnaK*, or fts-for and fts-rev for *ftsZ*, and inserted into pUT18 at HindIII and BamHI, fusing the C-terminus of the target protein to the N-terminus of the T18 domain. The plasmids constructed are listed in Table S4.

### 2.2. Growth Media and Conditions

Cells were exponentially grown in LB [24] and ABTGcasa [25] at 30 °C, 37 °C and 42 °C. Fifty μg/mL of kanamycin, 30 μg/mL of chloramphenicol, 15 μg/mL of tetracycline and 50 μg/mL of ampicillin were added when required for selection.

### 2.3. Fluorescence Microscopy

Exponentially growing cells of the W3110 strain and its derivatives carrying the different plasmids (see text in details) in ABTGcasa or LB medium at 30 °C, 37 °C or 42 °C were collected, washed, resuspended in 1× TE buffer and fixed in 70% ethanol. Expression of the GFP fusions was induced by addition of 0.1 mM isopropyl β-D-1-thiogalactopyranoside (IPTG) in the media. Cells were observed by Axio Imager A2 fluorescence microscopy (Carl Zeiss, Oberkochen, Germany) after 30 min of staining in Hoechst33258, which dyes DNA specifically, using chroma filter sets 13 for visualizing GFP, 43 for *mCherry* and 49 for Hoechst33258. Images were photographed using AxioCam MRc5 camera (Carl Zeiss) and analyzed with the Axio Vision Rel. 4.8 software (Carl Zeiss).

### 2.4. Total RNA Extraction

Total RNAs were isolated from the W3110 strain and its derivative cells using Trizol reagent (Transgen, BJ, China) kit following the manufacturer’s instructions. RNA integrity was verified by electrophoresis on a 1.2% agarose gel containing formaldehyde, and post stained with 1.0 μg/mL ethidium bromide. The 23S/16S ratios of all samples measured using an AgilentBio analyser (Agilent Technologies, CA, USA) were found to be about 2:1. RNA purity was determined using the NanoDrop 2000C spectrophotometer (Thermo Scientific™, Watham, MA, USA) by finding the A260/A230 and A260/A280 ratios. The A260/A280 ratios of all samples were 1.9–2.1 and the A260/A230 ratios were 2.0–2.1. Both integrity and purity of the RNA samples met the requirements for transcriptional microarray and the reverse transcriptional quantitative PCR (RT-qPCR) analysis [26].

### 2.5. Global Transcription Microarray Analysis

W3110∆*torRdnaC2*/pTorR-GFP cells were grown to exponential phase in ABTGcasa medium with or without TorR-GFP expression at 30 °C (the permissive temperature), and then the cells were up-shifted to 42 °C (the non-permissive temperature) for 2 h to synchronize the cells. The synchronized cells were down-shifted to 30 °C and then cell samples were collected at the 0 and 45 min time points respectively (Figure 2a). The Affymetrix GeneChip *E. coli* Genome 2.0 array (Affymetrix, Santa Clara, CA, USA) includes approximately 10,000 probe sets for all 20,366 genes present in *E. coli* K12 (MG1655), O157:H7-EDL933, O157:H7-Sakai and CFT073 strains and over 700 intergenic regions. For MG1655, probe sets are tiled to detect 4,358 open reading frames. The raw data was analysed by GeneChip Operating Software (Affymetrix) and the expression value for each gene of W3110∆*torRdnaC2*/pTorR-GFP cells at the 45 min time point was divided by the expression value at the 0 min in the presence of TorR-GFP. The same calculation between the values at the 45 min and 0 min time points was performed in the absence of TorR-GFP. Further, the two-set calculations were compared to screen the genes which could be controlled by the TorR protein in a cell-cycle-dependent manner. Two individual experiments were performed for validation. In detection analysis, we obtained “present” calls for between 3500 and 4000 genes, using target signal of 100 units. For comparison analysis, we filtered for “present” calls in both arrays. Detection and change are calculated as described in a previous report [27]. The data sets were validated by comparing the transcription of known genes, expression of all genes in an operon, and comparing two replicates. The “absent” calls for most genes from the three pathogenic strains mentioned above were also served as negative controls. The data discussed in this publication have been deposited in NCBI’s Gene Expression Omnibus and are accessible through GEO Series accession number GSE72525 [28].

### 2.6. Relative Quantitative Real-Time PCR

For the RT-qPCR measurement, total RNAs were isolated from W3110∆*torRdnaC2*/pTorR-GFP cells collected at 0, 15, 25, 35, 45 and 55 min after synchronization (Figure 2a) with or without induction of TorR-GFP expression by IPTG. Complementary DNA (cDNA) synthesis was performed with 5 μg of total RNA as template in a 25 μL reaction mixture including 4 μmol/L of each primer using the PrimeScript™ 1st Strand cDNA Synthesis Kit (TaKaRa, Otsu, Japan) as described in the manufacturer’s protocol. Cell-cycle-dependent expression levels of each gene chosen were measured by RT-qPCR using the corresponding primer pair listed in Table S5 and the cDNA as template. The primers for RT-qPCR were designed using the Primer Express v3.0 (Applied Biosystems, Foster City, CA, USA), meeting the requirements with Tm values of 55–62 °C and products shorter than 200 bp. SYBR^®^*Premix ExTaq*^TM^II kit (TliRNaseH Plus) (TaKaRa) was used for the RT-qPCR assay, and the reactions were performed in an Opticon 3 Real-Time PCR System (BioRad, Hercules, CA, USA) using the default cycling conditions (40 cycles of 95 °C for 5 s, 60 °C for 30 s after an initial incubation at 95 °C for 30 s). Melting curves were run immediately after the last cycle by reading the value at every increase of one degree for 10 s with the temperature ramping from 65 °C to 95 °C to exclude any influence of primer dimers. The Ct value for each reaction was automatically obtained, and the relative expression of target gene was calculated using the formula 2^(−ΔΔ*C*t)^. Expression values were normalized to that of the *rplO* gene as a reference. The experiments were repeated three times with two technical replicates for each experiment.

### 2.7. Immunoprecipitation by GFP-Trap

Exponentially growing cells in ABTGcasa medium at 37 °C were harvested at OD_450_ = 0.4, and lysed on ice in 5 mL lysis buffer containing 50 mmol/L Tris/Cl (pH8), 25 mmol/L NaCl, 2 mmol/L EDTA, 0.1% Triton X-100, 1M PMSF, 10 mg/mL lysozyme and 1× protease inhibitor cocktail (Roche, Mannheim, Germany). The resultant lysate was shaken at 37 °C in a water bath for 30 min, frozen in liquid nitrogen and thawed by incubation at 37 °C for 15 min. The lysate was sonicated on ice for 20 min until the sample was no longer viscous. The supernatant was collected by centrifugation at 20,000× *g* for 15 min, and transferred to a pre-cooled 1.5 mL tube on ice. GFP-trap^®^ beads (Chromotek, Planegg, Germany) were equilibrated in dilution buffer containing 10 mmol/L Tris/Cl, pH7.5, 150 mmol/L NaCl, 2 mmol/L MgCl_2_, 1 mmol/L PMSF (Beyotime, Beijing, China) and 1× protease inhibitor cocktail (Roche). The equilibrated 30 μL of bead slurry was washed in 500 μL ice-cold dilution buffer for three times at 4 °C by centrifugations and resuspensions. The cell lysate was added to the equilibrated GFP-trap^®^ beads and incubated with gentle end-to-end rotations for 12 h at 4 °C. The beads containing proteins were collected and washed two times in 500 μL ice-cold dilution buffer. The proteins bound on beads were eluted with 2× SDS sample buffer containing 2% SDS, 20% glycerol, 2% β-mercaptoethanol, 0.5 M Tris/Cl, pH 6.8 and 0.1 mg/mL bromphenol blue dye, for sodium dodecyl sulfate polyacrylamide gel electrophoresis (SDS-PAGE) and mass spectrometry analysis.

### 2.8. SDS-PAGE and Mass Spectrometry Analysis

The eluted proteins were separated by SDS-PAGE, stained in Coomassie blue for 30 min with gentle shaking, following destaining in buffer containing 40% (*v*/*v*) methanol, 10% (*v*/*v*) acetic acid for several hours with gentle shaking. The protein bands were cut and then analyzed by mass spectrometry. Peptides resulting from proteolytic digestion of protein gel bands were analyzed by tandem mass spectrometry using a Q Exactive LC/MS (Thermo Scientific) Liquid Chromatography-Tandem Mass Spectrometry to give a peptide mass fingerprint and additional peptide sequence information which was searched against *E. coli* K12-W3110 database using the Mascot search engine from Matrix Science to identify the protein as described previously [29]. Western blotting probed with GFP antibody was performed as described previously [30].

### 2.9. Bacterial Two Hybrid Analysis

Plasmids required for the bacterial two hybrid system (BCATH) are described above in the ‘Bacterial strains and plasmids’ section. Cells for BACTH assays were cultured at 30 °C for about 30 h on LB agar plates supplemented with ampicillin (100 μg/mL), kanamycin (50 μg/mL), 0.5 mM IPTG and chromogenic substrate X-gal (40 μg/mL).

### 2.10. Site-Directed Mutagenesis

Point mutation was generated using the site-directed mutagenesis kit as described previously [31]. The multi-mutations (S178N, R179H, T198N, L210P, G222A) in pTorR-GFP were, respectively, prepared by PCR using the pTorR-GFP as template and pairs of primers which are listed in Table S5. The deletion the C- or N-terminus of TorR, MreB or DnaK was, respectively, generated by PCR using the pTorR-GFP, pKNT-*torR*, pUT-*mreB* or pUT-*dnaK* plasmid (Table S4) as template and corresponded pair of primers as listed in Table S5.

## 3. Results

### 3.1. The TorR Protein Is Localized at the Old Cell Pole as a Functional Focus

**Figure 1:**
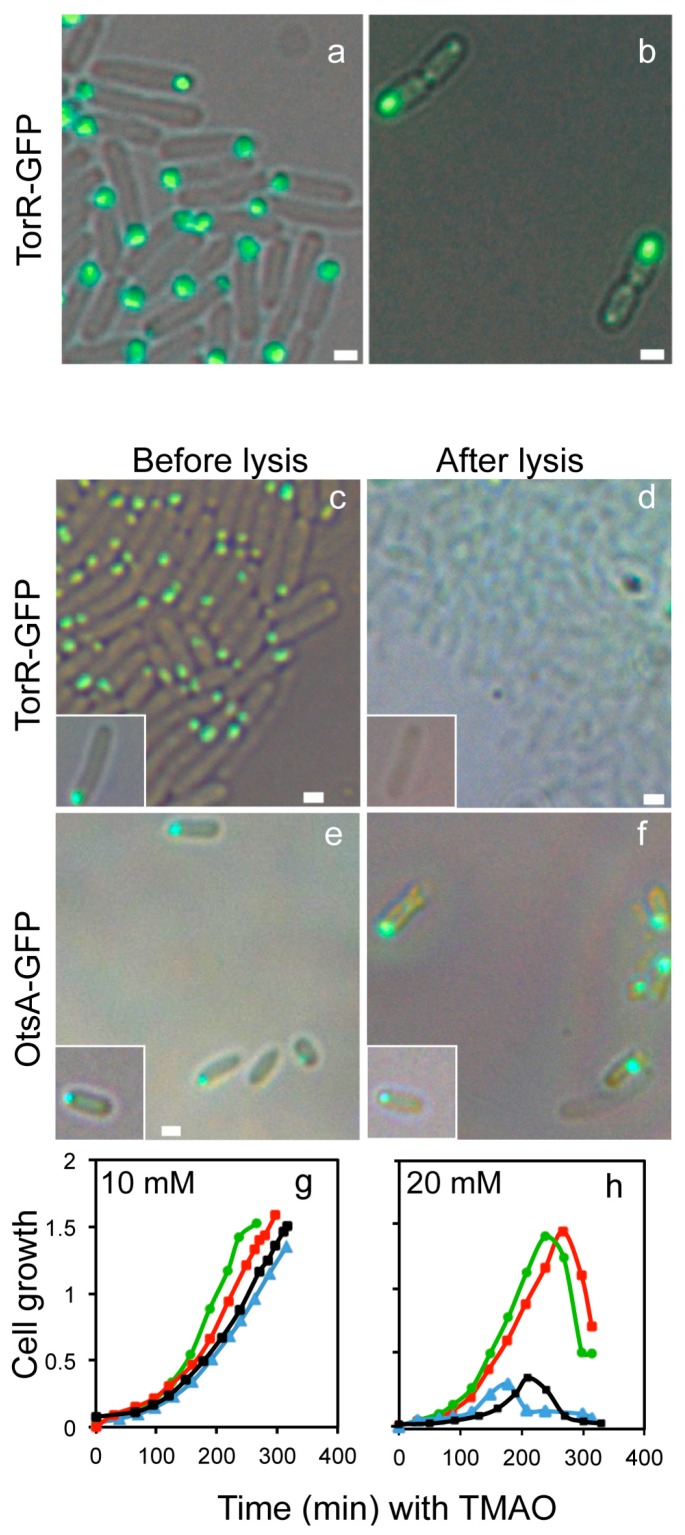
The TorR-green fluorescent protein (GFP) fusion localizes at the old cell poles as a focus. Exponentially growing cells in ABTGcasa containing isopropyl β-D-1-thiogalactopyranoside (IPTG) to induce expression of TorR-GFP from the pTorR-GFP plasmid at 37 °C were fixed in 70% ethanol. The TorR-GFP fusion was then visualized by fluorescence microscopy. (**a**,**b**) represent cells of W3110Δ*torR*/pTorR-GFP with green TorR-GFP fusion. Cells expressing TorR-GFP (**c**,**d**) or OtsA-GFP (**e**,**f**) were exponentially grown and visualized in 1.2% agarose pads of ABTGcasa medium before (**c**,**e**) and after (**d**,**f**) cell lysis (2 mg/mL lysozyme, 0.1% Triton X-100, and 10 mM EDTA). The insets indicate the same cell before and after cell lysis. The scale bars represent 1 μm. Exponentially growing wild-type W3110 (green), W3110Δ*torR* (blue) and W3110Δ*torR*/pTorR-GFP cells with IPTG (red) and without IPTG (black) were diluted 10 times in ABTGcasa with 10 mM (**g**) or 20 mM Trimethylamine N-oxide (TMAO) (**h**), and growth curves were monitored. The X axis presents time in TMAO and Y axis indicates cell growth measured by cell density (OD_450_). The values are the average of three experiments.

To visualize the subcellular localization of the TorR-GFP fusion protein, W3110Δ*torR* cells carrying plasmid pTorR-GFP were exponentially grown in ABTGcasa medium at 37 °C. In the cells, there is no chromosomally encoded TorR to compete for the target sites with TorR-GFP. Expression of the TorR-GFP fusion protein was induced by treating the cells with IPTG for 2–3 generations before sampling. The cells were fixed in 70% ethanol and visualized in a Axio Imager A2 microscope (Carl Zeiss). TorR-GFP localized in a focus at one cell pole (Figure 1a). In septated cells there was only one TorR-GFP focus at one of the old poles (Figure 1b), indicating that TorR-GFP targets the old pole and that a new TorR-GFP focus forms at the old poles of the new-born cells after cytokinesis. In agreement with the result, it was found that the mYFP-tagged TorR clearly clustered at one pole of the *E. coli* cells [32].

Misfolded or overexpressed fusion proteins frequently form inclusion bodies and aggregate at the poles due to nucleoid occlusion from the middle of the cells [33,34]. Inclusion bodies are insoluble protein aggregates which remain aggregated upon cell lysis [35]. To rule out the possibility that the TorR focus represents inclusion bodies, live W3110Δ*torR* cells expressing TorR-GFP were mounted onto an agarose pad of ABTGcasa medium with lysozyme for cell lysis. As a control, cells expressing OtsA-GFP were included in the experiments since overexpression of OtsA leads to rapid formation of inclusion bodies which remain visible as insoluble aggregates after cell lysis [36]. The fluorescent protein fusions in the cells before and after lysis were visualized by fluorescence microscopy. As expected, the OtsA-GFP fluorescent inclusion bodies remained visible after the cells had lysed (Figure 1e,f) whereas the fluorescence signal from TorR-GFP completely disappeared as soon as the cells lysed (Figure 1c,d), suggesting that the TorR-GFP foci are soluble. To further confirm that the TorR-GFP focus is not inclusion bodies, we co-transformed the W3110Δ*torR* cells with the pTorR-GFP and pIbpA-mCherry plasmids. The pIbpA-mCherry plasmid encodes the IbpA-mCherry fusion under control of the *ibpA* promoter [37] and IbpA-mCherry is a marker for inclusion bodies [38]. It was found that the TorR-GFP and IbpA-mCherry foci did not overlap (Figure S1a,b). The TorR-GFP foci were found to be soluble while the IbpA-mCherry foci remained visible as inclusion bodies after cell lysis (Figure S1e,f). Also, the co-expressed OtsA-GFP and TorR-mCherry foci were found not to overlap. The foci rather occupied distinct regions of the cell pole (Figure S1c,d). Taken together, the TorR polar focus is a protein structure that is distinct from inclusion bodies.

Further, to confirm that TorR-GFP fusion protein is functional *in vivo*, the wild-type W3110 cells and its Δ*torR* and Δ*torR*/pTorR-GFP derivatives were grown in ABTGcasa medium with TMAO, and the growth curve of the bacteria was monitored. As described in the Introduction section, TorR generates the TMAO reductase [17] and the reductase reduces TMAO to trimethylamine (TMA) [39]. The presence of TMA increases the pH of the aerobic culture to facilitate growth [40]. In the presence of low concentration of TMAO (Figure 1g), Δ*torR* cells showed a slight growth defect compared with the wild-type cells. However, at high concentration of TMAO the growth defect for Δ*torR* cells was dramatic and it was reversed by the presence of either TorR-GFP fusion protein (Figure 1h) or the wild-type TorR protein (Figure S1g,h). The results indicate that the TorR-GFP fusion protein reduces TMAO to TMA, demonstrating that the TorR-GFP fusion is biologically functional.

### 3.2. TorR Regulates Transcription of Several Genes in a Cell-Cycle-Dependent Manner

The TorR protein is the response regulator of the TorS/TorR two-component signal transduction system [41] that regulates transcription of several genes [42]. How can TorR manage to regulate gene transcriptions from a polar localization? There might be two possibilities: (i) TorR-GFP could be dynamic in subcellular localization so that TorR interacts with its target genes from time to time; (ii) interaction between TorR-GFP and the target genes could be cell-cycle-dependent. To check the first possibility, we observed the TorR-GFP focus in live cells for several minutes and did not observe a change in subcellular localization of TorR-GFP (Movie S1).

**Figure 2:**
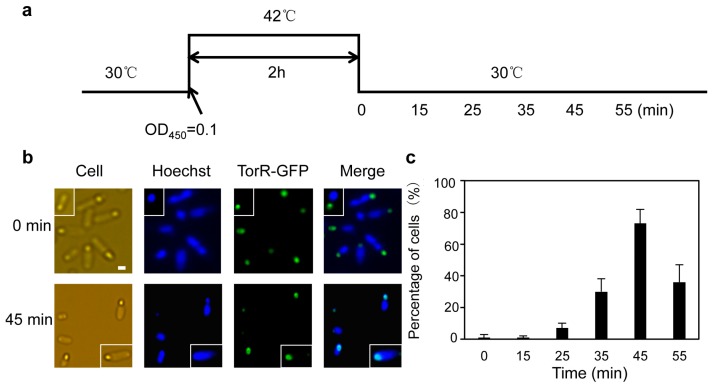
The TorR-GFP focus co-localizes with the nucleoid in a cell-cycle-dependent fashion. (**a**) Exponentially growing W3110Δ*torRdnaC2* cells carrying pTorR-GFP in ABTGcasa medium with IPTG for expression of TorR-GFP were shifted from the permissive temperature (30 °C) up to the non-permissive temperature (42 °C). After 2 h incubation at 42 °C, the synchronized cells were shifted down to 30 °C, and sampled at the time points indicated and fixed in 70% ethanol; (**b**) Subcellular localizations of TorR-GFP and the nucleoid were visualized by Axio Imager A2 fluorescence microscope (Carl Zeiss) and AxioCam MRC5 camera after 30 min staining in Hoechst33258 which dyes DNA specifically. The TorR-GFP focus is in green, the nucleoid is in blue, the merged image of TorR-GFP focus with the nucleoid is in cyan. The time points at which cells were collected are as indicated on the left; (**c**) The percentage (%) of cells with co-localized TorR-GFP focus with the nucleoid was counted and shown as a function of time (min) at 30 °C. More than 100 cells were included for each calculation at each time point. The values are the average of three individual experiments and the standard errors are given as the error bars.

To monitor if TorR interacts with its target genes in a specific period of time during the cell cycle, we synchronized the W3110Δ*torR* cells harbouring the plasmid pTorR-GFP, using a *dnaC2* mutant. The *dnaC2* cells can be synchronized at a stage before initiation of chromosome replication by a temperature shift [43,44]. Exponentially growing W3110Δ*torRdnaC2* cells carrying pTorR-GFP were shifted from the permissive temperature (30 °C) to the non-permissive temperature (42 °C) (Figure 2a). New initiation of replication was then inhibited, and all cells had only one fully replicated chromosome after 120 min as a result of ongoing replication and cell division (Figure S2a). When the culture was shifted down to the permissive temperature all cells initiated replication from *oriC* in a synchronous manner. To investigate a possible co-localization of TorR-GFP with the nucleoid the subcellular localization of TorR-GFP focus and the nucleoid were visualized by fluorescence microscopy. Treatment of the cells with lysozyme led to disappearance of TorR-GFP foci, indicating that the TorR-GFP fusion protein in the temperature-shift treatment is soluble (Figure S2b). As shown in Figure 2b, the green focus of TorR-GFP and the blue Hoechst-stained DNA were separated in most of the cells tested at the 0 min time point and they overlapped 45 min after the shift-down to the permissive temperature (Figure 2b). As the cell cycle progressed, the proportion of cells with co-localized TorR-GFP focus and nucleoid increased gradually, from 30% at the 35 min time point to a maximum of 73% at the 45 min time point. At the 55 min time point, the proportion of cells sharply dropped to 36% (Figure 2c). The results indicate that co-localization of TorR and the nucleoid is cell-cycle-dependent, and that co-localization reaches a peak 45 min after the cells are released from initiation-arrest. The generation time at the permissive temperature was about 70 min in the growth condition used. The wild-type *E. coli* B/r strain has a fixed period of 40 min for replication and a time of 20 min for cell division with a growth-rate-dependent period from cell birth to initiation of replication [45]. Together, the data and previous findings imply that co-localization of TorR with its target genes occurs after the end of replication, at which TorR may interact with its target genes. The co-localization of TorR and the nucleiod is likely due to chromosome segregation toward cell poles, allowing cell septation.

To address why TorR co-localizes with the nucleoid in a cell-cycle-dependent fashion, we performed an *E. coli* Genome GeneChip microarray assay in the presence or absence of TorR protein as a function of the cell cycle. The expression level of each gene at the 45 min relative to the 0 min time point was calculated by finding the ratio of values at 45 min to that at 0 min. The present and absent calls were validated as described previously [27]. We found that the expression of 29 genes was down-regulated while one gene (*ftn*) was up-regulated in the presence of TorR-GFP at the 45 min relative to the 0 min time point (Table S1). On the contrary, the expression of these genes was not changed in the absence of TorR-GFP (Table S1). The results suggest that the 30 genes are regulated by TorR in a cell-cycle-dependent manner. These genes encode proteins involved in Fe-S cluster assembly (*sufABCDSE* operon), iron transportation (*fepA*, *fepC*, *fes, fhuB* and *fhuE*) and signal transduction (*fecAR*). In agreement with the findings here, expressions of *fecA*, *fepC*, *ybjH*, *sufD* were increased, and that of the *ftn* gene was decreased in Δ*torR*Δ*torS*Δ*torCAD* cells [46].

**Figure 3:**
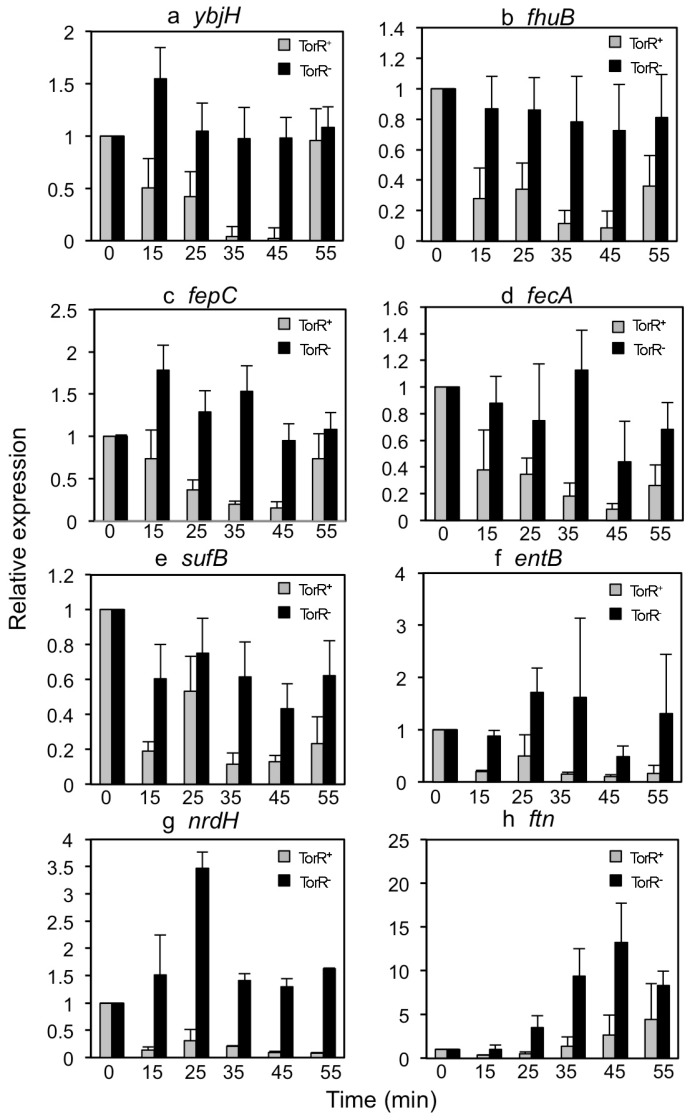
Cell-cycle-dependent co-localization of TorR-GFP focus with the nucleoid affecting expression of several genes. Synchronization, induction of TorR-GFP expression, and preparation of W3110Δ*torRdnaC2* cells carrying pTorR-GFP were as described in the legend of Figure 2. Total RNA isolation, complementary DNA (cDNA) synthesis and performance of reverse transcriptional quantitative PCR (RT-qPCR) were as described in Materials and Methods. Each gene expression was normalized using the Ct value corresponding to the *E. coli rplO* gene as an internal reference and the relative expression at every time point to the 0 min was calculated. The expression values are the mean of three individual experiments and the standard errors are given as the error bars. The X-axis indicates the time intervals after shift-down of the culture to the permissive temperature (30 °C) and the Y-axis represents the relative expression of each gene. The grey bars are for expressions of genes in the cells with TorR-GFP and the black bars are for that in the cells without TorR-GFP. The values are the average of three experiments. The error bars are as indicated. All the genes analyzed are as indicated (**a**–**h**).

Next, to validate the data obtained from transcriptional array analysis, we performed RT-qPCR. Synchronization of W3110Δ*torRdnaC2* cells containing pTorR-GFP was as described in Figure 2a. RNA isolation and cDNA synthesis were as mentioned above. For analysis of the kinetics of transcription during the cell cycle, eight genes were randomly chosen from 30 genes with changes in expression as a function of the cell cycle in the presence of TorR-GFP. As shown in Figure 3, expressions of five genes (*ybjH*, *fhuB*, *fepC*, *fecA* and *sufB*) out of eight tested in the presence of TorR-GFP relative to that at the 0 min gradually decreased up until 45 min and increased sharply at the 55 min time point. The fluctuations in expression of the genes accordingly responded to changes in the number of cells with the co-localized TorR and the nucleoid. The expressions reached the lowest level at the 45 min time point when TorR co-localized with the nucleoid in 73% of cells, and were increased at the 55 min time point when TorR overlapped with the nucleoid only in 36% of cells (Figure 2c and Figure 3a,d). Yet, expressions of the five genes were not clearly oscillating with the progression of cell cycle in the absence of TorR-GFP fusion (Figure 3a–e). Expression of *entB* and *nrdH* was repressed in the presence but not in the absence of TorR-GFP, and the repression was not clearly fluctuating during the cell cycle (Figure 3f,g). Interestingly, expression of the *ftn* gene was gradually increased relative to the 0 min time point in both the presence and absence of TorR-GFP. However, it should be noted that the *ftn* expression was higher in the absence compared to that in the presence of TorR-GFP (Figure 3h). The result indicates that the *ftn* gene is regulated by an unknown factor which functions in a cell-cycle-dependent manner with the assistance of TorR. The findings are in accordance with the results from transcriptional microarray analysis, suggesting that the microarray data is reliable and reproducible. The same cell-cycle-dependent fluctuations in expressions of *ybjH*, *fhuB* and *fepC* were found in the *dnaC2* mutant but not in Δ*torRdnaC2* cell (Figure S3), the former containing the wild-type chromosomal *torR* gene. The results again confirm that the TorR-GFP fusion is functional. Together, the results allow us to conclude that TorR regulates the expression of several genes by interacting with its target genes in a cell-cycle-dependent fashion.

### 3.3. TorR Interacts with MreB and DnaK for Targeting the Old Cell Poles

We have shown that the TorR protein forms a focus at the old cell pole and co-localizes with the nucleoid in a short period of the cell cycle. This short-time co-localization of TorR and the nucleoid ensures that TorR represses or activates the target genes in a given short period of the cell cycle. We then investigated how TorR localizes as a focus at the old cell pole. To address the question, we pulled down the proteins that bound to TorR using the GFP-Trap technique [47]. The cell lysate containing TorR-GFP were mixed with GFP-Trap beads and the proteins bound were characterized by mass spectrometry. Also, the cell lysate having YfiF-GFP was included in the experiment serving as controls. Taking the function and molecular weight (found in the SDS-PAGE gel) (Figure S4) of the proteins into consideration, the DnaK, MreB, OmpA, OmpR, OmpT and several other proteins were identified (Table S2). These proteins may interact with TorR in the cell extract and are likely partners for the polar localization of TorR.

**Figure 4:**
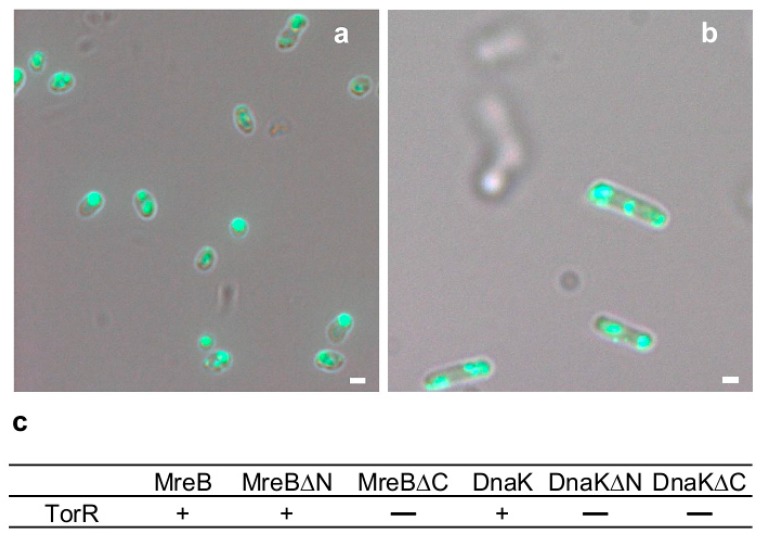
The MreB and DnaK proteins interact with TorR and are required for localization of TorR-GFP at the old poles of cells. (**a**) Exponentially growing W3110Δ*torR*/pTorR-GFP cells in ABTGcasa with IPTG at 37 °C were treated with A22 (10 μg/mL) for 6 h to inhibit MreB, and then fixed in 70% ethanol. The subcellular localization of TorR-GFP was visualized by fluorescence microscopy. The scale bar represents 1 μm; (**b**) Growth condition and TorR-GFP visualization in exponentially growing W3110Δ*dnaK*/pTorR-GFP cells were as described in the legend to (**a**). The scale bar represents 1 μm; (**c**) BTH101 cells were co-transformed with a mixture of pKNT-*torR* and pUT-*mreB* plasmids for testing TorR-MreB interaction, pKNT-*torR* and pUT-*dnaK* for TorR-DnaK, pKNT-*torR* and pUT-*mreB*_Δ2-90_ for TorR-MreBΔN, pKNT-*torR* and pUT-*mreB*_Δ180-281_ for TorR-MreBΔC, pKNT-*torR* and pUT-*dnaK*_Δ1-100_ for TorR-DnaKΔN, or pKNT-*torR* and pUT-*dnaK*_Δ510-639_ for TorR-DnaKΔC interaction, plated on LB agar plates containing X-gal, IPTG, and required antibiotics, incubated at 30 °C for 30 h. **+** stands for protein–protein interaction as shown (blue colonies) in Figure S7—presents no protein–protein interaction as indicated (white colonies).

To further evaluate these possible TorR partners, Δ*dnaK*, Δ*ompA*, Δ*ompR*, Δ*ompT*, Δ*pstB*, Δ*ybgI* and Δ*ycfH* mutants were exponentially grown with expression of TorR-GFP. Subsequently, the subcellular localization of TorR-GFP was investigated in each mutant by fluorescence microscopy. In the Δ*dnaK* cells, TorR-GFP formed several punctae instead of one focus at one pole (Figure 4b), suggesting that localization of TorR at the old pole of cell requires DnaK. In contrast, in another six mutants tested, it displayed a single polar TorR-GFP focus (Figure S5). DnaK is an Hsp70 (heat shock protein) chaperone, being involved in the folding of nascent polypeptide chains [48] and protein export [49].

The MreB protein is involved in polar protein localization, cell shape determination, cell division, chromosome movement and polar localization of the chromosomal origin sequence [50,51]. Thus, it is possible that MreB contributes to the polar localization of TorR. MreB is an actin homolog in rod-shaped prokaryotic cells. In *E. coli* MreB forms helical filaments along the long axis of the cells [52] and is essential for cell survival. To inhibit MreB function, *S*-(3,4-Dichlorobenzyl) isothiourea (A22) was used, which is a well-known inhibitor of MreB, interacting directly with MreB as a competitive inhibitor of ATP [53]. Exponentially growing W3110Δ*torR* cells harboring pTorR-GFP were treated with A22 for 6 h, the subcellular localization of TorR-GFP was visualized by fluorescence microscopy. Approximately 71% (Figure S6b) of the cells contained several puncta instead of a single TorR-GFP focus (Figure 4a), suggesting that MreB is required for localization of TorR at the old pole of cell. In agreement with the findings, overexpression of MreB from a plasmid also led to formation of spherical cells and mislocalization of TorR-GFP instead of a polar localized focus (Figure S6a).

To investigate a possible interaction of TorR with MreB or DnaK, the bacterial two-hybrid assay (BACTH) [23] was utilized. When two proteins interact in the bacterial two-hybrid system, the *lacZ* gene is expressed in a cAMP/CRP (cAMP receptor protein)-dependent mode [23], forming blue colonies on LB plates containing X-gal (see Materials and Methods for further details). The cells expressing TorR-MreB and TorR-DnaK gave rise to blue colonies (Figure S7) whereas the negative control cell colonies remained white (Figure S7). The results demonstrate that TorR interacts with MreB and DnaK in vivo (Figure 4c), suggesting that direct interaction of TorR with MreB and DnaK may be required for the polar localization of TorR. In order to determine the domains of MreB or DnaK responsible for interaction with TorR, we deleted their N- or C-terminus, resulting in MreBΔ2-90 (N-terminal) and MreBΔ180-281 (C-terminal), or DnaKΔ1-100 (N-terminal) and DnaKΔ510-639 (C-terminal) mutant proteins. Using the bacterial two-hybrid system mentioned above, we found that deletion of the C-terminus of MreB abolished its interaction with TorR (Figure 4c and Figure S7), but lacking the N-terminus did not affect the interaction (Figure 4c and Figure S7), suggesting that the C-terminus of MreB is required for the MreB-TorR interaction. For the DnaK-TorR interaction, either deletion of the N- or C-terminus of DnaK led to the loss of interaction (Figure 4c and Figure S7), suggesting that the full length DnaK protein is necessary for the interaction.

Also, it was found that the fused proteins YbgI-GFP, OmpT-GFP, MalF-GFP, PstB-GFP, YcfH-GFP and OmpR-GFP localized at the cell poles, and OmpA-GFP fusions were around the outer membrane (Figure S8). The polar localization of these proteins may contribute to the polar localization of TorR with unknown mechanisms. GpmA-GFP fusion was diffusely distributed all over the cytoplasm (Figure S8), suggesting that they may not be involved in the polar localization of TorR.

### 3.4. CCCP Treatment Affects the Polar Localization of TorR and Expression of the Target Genes

**Figure 5:**
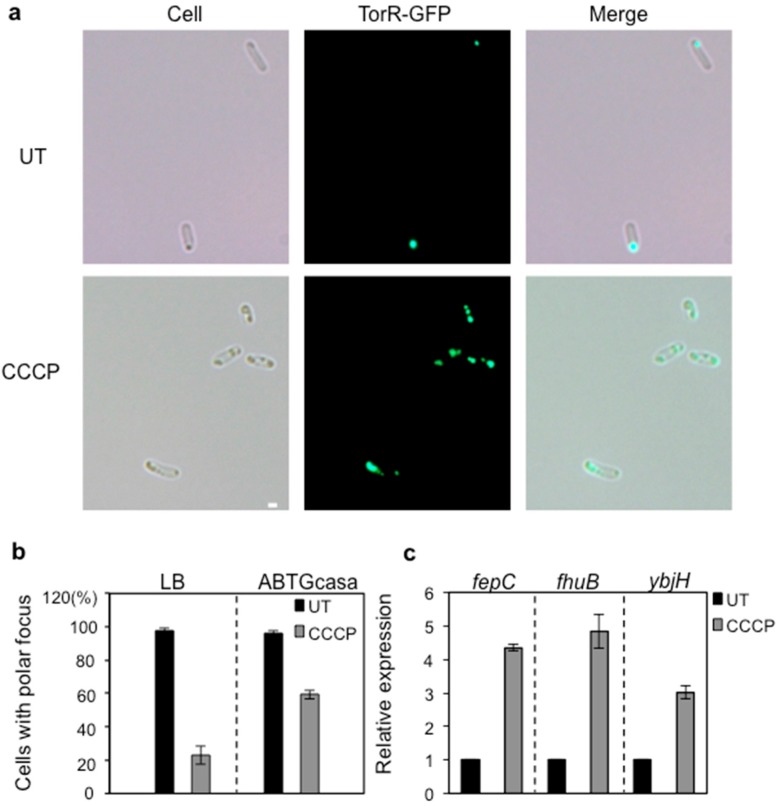
Polar localization and regulatory function of TorR-GFP on gene expression are dependent on ATP. (**a**) W3110Δ*torR*/pTorR-GFP cells were exponentially grown in LB with IPTG for expression of TorR-GFP at 37 °C. The cells were treated or untreated with 100 μM carbonyl cyanide 3-chlorophenylhydrazone (CCCP) at OD_450_ = 0.25 for 5 min to abolish ATP synthesis, and fixed in 70% ethanol. TorR-GFP visualization was as described in the legend to Figure 1. The top panels are for cells untreated, shown as UT for short; lower panels are for cells treated with CCCP. The scale bar represents 1 μm; (**b**) Exponentially growing W3110Δ*torR*/pTorR-GFP cells in LB or ABTGcasa at 37 °C were treated and analyzed as described in the legend to (**a**). The percentage of cells with a single focus or multi puncta of TorR-GFP was calculated. About 100 cells were included in each calculation. The values are average of three individual experiments and standard errors are given as the error bars; (**c**) Expression of the *fepC*, *fhuB* and *ybjH* genes in synchronized W3110Δ*torRdnaC2*/pTorR-GFP cells was determined by RT-qPCR as described in the legend to Figure 3. At the 45 min time point after release from the non-permissive temperature as described in the legend to Figure 2, the cells with or without treatment of CCCP (10 min) were collected for the analysis. The values are the average of three individual experiments and standard errors are given as the error bars. The grey bars are for gene expression in the CCCP-treated cells and the black bars are for gene expression in the untreated cells.

To understand whether delivery of TorR protein to the old poles of cells needs the energy from ATP, we used the protonophore CCCP (carbonyl cyanide 3-chlorophenylhydrazone) to abolish the proton motive force (PMF) and subsequently the formation of ATP [54]. W3110Δ*torR* cells harboring pTorR-GFP were exponentially grown in LB or ABTGcasa containing IPTG with or without CCCP treatment, and the subcellular localization of TorR-GFP was investigated. We found that 77% and 41% cells had several punctae of different sizes of TorR-GFP after CCCP treatment when the cells were grown in LB and ABTGcasa medium, respectively, compared to 2%–4% in cells without CCCP treatment (Figure 5a,b). It should be noted that 23% or 59% cells had a TorR-GFP focus at one pole of cells in LB or ABTGcasa after CCCP treatment (Figure 5b).

To investigate whether the defect in polar localization of TorR due to CCCP treatment affects transcription of the target genes, we chose the *ybjH*, *fhuB* and *fepC* genes since these genes were shown to be regulated by TorR in a cell-cycle-dependent manner (Figure 3a–c). Transcription of the three genes was measured by RT-qPCR in the synchronized cells grown in ABTGcasa after CCCP treatment. Relative to the expression level of the *fepC*, *fhuB* or *ybjH* gene in cells untreated with CCCP, the expression was increased in cells treated with the drug. In particular, expression of the *fhuB* gene was increased by about five-fold in the cells treated with CCCP (Figure 5c). The results suggest that ATP is required for delivering the TorR protein to the cell pole and for subsequent regulation of gene expression.

### 3.5. FtsZ Affects the TorR Foci Formation

**Figure 6:**
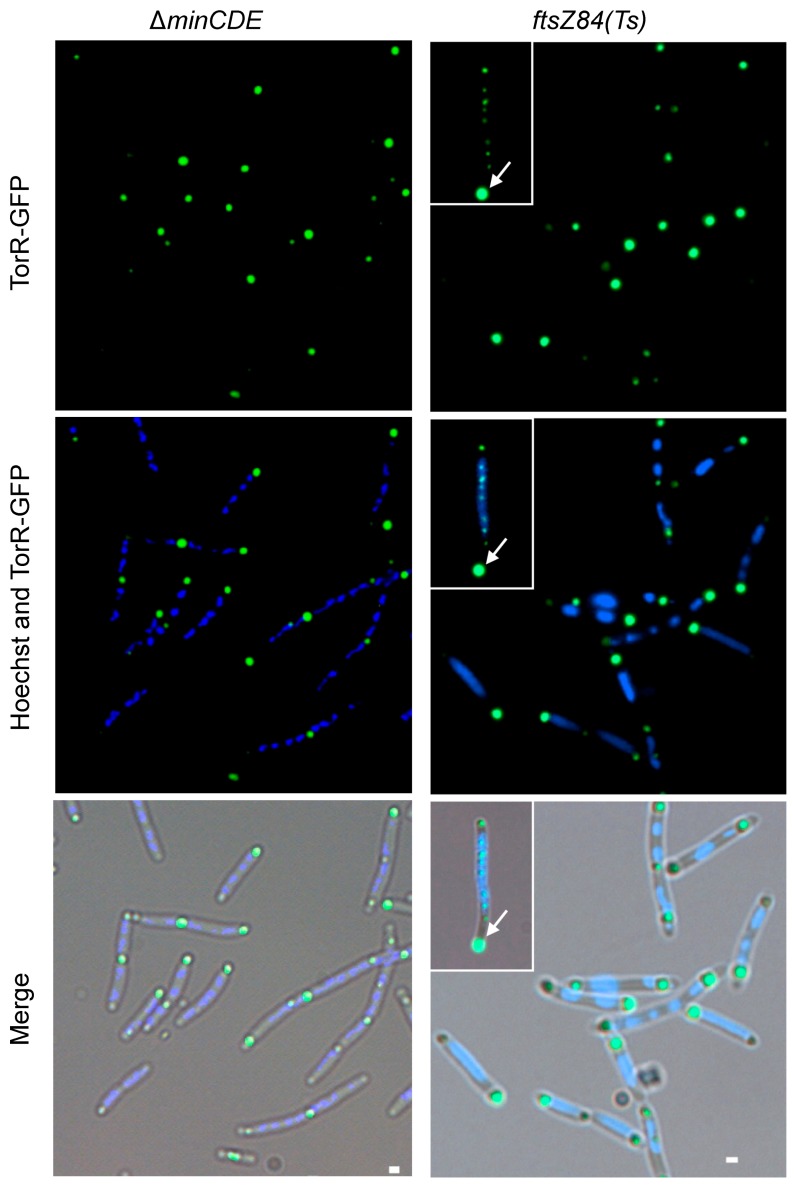
Formation of a ‘normal’ TorR-GFP focus depends on FtsZ and the MinCDE proteins. Exponentially growing W3110Δ*torR*Δ*minCDE*/pTorR-GFP cells in ABTGcasa with IPTG at 37 °C were collected. Subcellular localization of TorR-GFP and the nucleoids were visualized by fluorescence microscopy after 30 min staining in Hoechst33258 (the left panels). To inactivate the thermosensitive FtsZ84 protein, exponentially growing W3110Δ*torRftsZ84(Ts)*/pTorR-GFP cells in ABTGcasa at 30 °C were up-shifted to the non-permissive temperature (42 °C) for 2 h, subsequently TorR-GFP and the nucleoids were visualized after staining with Hoechst33258. The arrow indicates the cell with an enlarged TorR-GFP focus and several small TorR-GFP foci during the time course at 42 °C. The scale bars represent 1 μm.

The FtsZ protein is an essential cell division protein which polymerizes to form a ring-structure, and the ring sets the site of division and serves as a scaffold for recruitment of other division proteins [55]. The placement and timing of the FtsZ ring assembly is a highly-regulated process. The MinCDE proteins ensure that the FtsZ ring, and hence the septum, is formed at mid-cell [56] by oscillating from pole-to-pole [57]. To see if MinCDE or FtsZ affect the polar localization of TorR, W3110Δ*torRminCDE* cells containing pTorR-GFP were exponentially grown at 37 °C. W3110Δ*torRftsZ*84 (Ts) cells harboring pTorR-GFP grown at 30 °C were shifted up to the nonpermissive temperature (42 °C) to inactivate the thermosensitive FtsZ84 protein for 2 h. Then, the distribution patterns of TorR-GFP focus or foci were investigated through fluorescence microscopy. In the absence of the MinCDE proteins, the cells were elongated and two to five TorR-GFP foci were observed along the long axis of the cells. The TorR-GFP foci were almost even in size (Figure 6), suggesting that deletion of MinCDE allows for the formation of several TorR-GFP foci in a cell. Interestingly, inactivation of the FtsZ84 mutant protein led to formation of many TorR-GFP foci which were uneven in size in the elongated cells, and the TorR-GFP focus at the cell pole was likely growing stronger as a function of time at the non-permissive temperature (Figure 6). The elongated cells with many ‘abnormal’ TorR foci (Figure 6) accounted for 22% of the cells scored whereas cells with only one focus or cells with two foci (one big at one pole and one tiny at the other pole) (Figure 6) accounted for 78% of the cells scored. It seems that the absence of FtsZ does not affect delivery of TorR to the pole but it affects the formation of ‘normal’ TorR foci. It should be noted that TorR was not found to interact with FtsZ in either the pull-down or bacterial two-hybrid experiments. It is likely that the assembly of FtsZ-ring is a signal for formation of a TorR-GFP focus since several ‘normal’ TorR foci formed in the ∆*minCDE* cells where the several FtsZ-rings can be assembled [58], but new ‘normal’ TorR focus did not appear when the FtsZ-ring cannot be formed due to inactivation of FtsZ84.

### 3.6. Interaction of TorR with MreB or DnaK is Responsible for Its Polar Localization and Subsequent Regulatory Effect on Gene Expression

**Figure 7:**
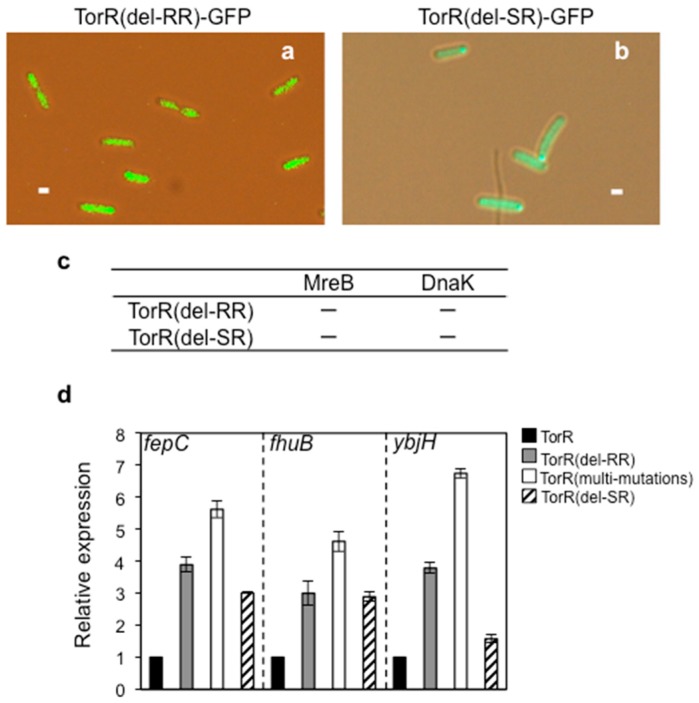
Deletion of the response regulator (RR) or signal receiver (SR) domain in the TorR protein abolishes its polymerization, polar localization and regulatory function on gene expression. (**a**) The RR domain of TorR was deleted in pTorR-GFP by site-directed mutagenesis kit, resulting in pTorR(del-RR)-GFP plasmid. Exponentially growing W3110Δ*torR/*pTorR(del-RR)-GFP cells in ABTGcasa with IPTG for expression of TorR(del-RR)-GFP were collected and TorR(del-RR)-GFP in the cells was visualized; (**b**) Deletion of the SR domain of TorR in pTorR-GFP and visualization of TorR(del-SR)-GFP were as described in the legend to (**a**); (**c**) Interaction of mutant TorR with MreB or DnaK was detected by the bacterial two hybrid system. The pKNT-*torR*(del-RR) and pUT-*mreB* plasmids were used to test for TorR(del-RR)-MreB interaction, pKNT-*torR*(del-RR) and pUT-*dnaK* for TorR(del-RR)-DnaK, pKNT-*torR*(del-SR) and pUT-*mreB* for TorR(del-SR)-MreB, pKNT-*torR*(del-SR) and pUT-*dnaK* for TorR(del-SR)-DnaK—represents no protein–protein interaction as shown (white colonies) in Figure S7; (**d**) Expressions of the *fepC*, *fhuB* and *ybjH* genes in synchronized W3110Δ*torRdnaC2*/pTorR(del-RR)-GFP, W3110Δ*torRdnaC2R*/pTorR(del-SR)-GFP, or W3110Δ*torRdnaC2*/pTorR(multi-mutations)-GFP cells in ABTGcasa were measured by RT-qPCR as described in the legend of Figure 3. The cells were synchronized and sampled at the 45 min time point as mentioned in the legend to Figure 2. The tested genes and mutated TorR-GFP fusions are as indicated. The values are the average of three individual experiments and the standard errors are given as the error bars.

A sequence comparison suggested that the N- and C-terminal parts of TorR protein could be the signal receiver (SR) and the response regulator (RR) (promoter targeting) domains, respectively [41]. To understand how the TorR protein localizes at the old poles and how it regulates expression of its target genes, we deleted the signal receiver (SR) or the response regulator (RR) of TorR by site-directed mutagenesis. Also, the truncated TorR-GFP proteins were in the expected molecular size range and were found to be expressed at the same level as the wild-type TorR-GFP protein which is intact (Figure S9a). The subcellular localization analysis showed that deletion of the SR domain (TorR(del-SR)-GFP) or the RR domain in TorR-GFP (TorR(del-RR)-GFP) destroyed its polar localization. The mutant TorR(del-SR)-GFP and TorR(del-RR)-GFP were found to be dispersed over the cytoplasm (Figure 7a,b). In addition, a mutant TorR (multi-mutations) protein with five point mutations (TorR S178N; R179H; T198N; L210P; G222A) in the RR domain was found to be in two foci, each of them occupying one pole of the cells (Figure S9b). The results indicate that the SR or RR domain of TorR is required for its polar localization.

To further analyze if the SR or RR domain is also needed for the protein-protein interaction, interaction of the mutant TorR with MreB or DnaK was investigated by the bacterial two-hybrid system. Deletion of the RR domain in TorR (TorR(del-RR)) abolished its interaction with MreB or DnaK (Figure 7c and Figure S7). Also the absence of SR domain in TorR (TorR(del-SR)) removed its ability to interact with MreB or DnaK (Figure 7c and Figure S7). These results suggest that either lacking the RR or SR domain abolishes the interaction of TorR with MreB or DnaK.

To see whether the interaction of TorR with MreB or DnaK is required for the regulatory effect of TorR on gene expression, we measured the expression of *fepC*, *fhuB* and *ybjH* genes, in the presence of the mutant TorR-GFP proteins mentioned above, in synchronized W3110Δ*torRdnaC2* cells. RT-qPCR analysis showed that the expression levels of the *fepC*, *fhuB* and *ybjH* genes were significantly increased in the SR- or RR-deleted TorR or multi-mutations in TorR (Figure 7d). These results allow us to propose that the interaction between TorR and MreB or DnaK is responsible for polymerization, polar localization and the subsequent regulatory effects of TorR on gene expression.

## 4. Discussion

### 4.1. The TorR Protein Targets the Old Cell Poles by Interacting with MreB and DnaK Proteins

We show that the TorR-GFP fusion localizes at the old cell pole (Figure 1a,b), and such polar localization requires MreB and DnaK with the assistance of ATP (Figure 4 and Figure 5). Interaction between TorR and MreB or DnaK is detected both in vitro and in vivo, and the interaction requires full length DnaK and the C-terminal domain of MreB (Figure 4). In *E. coli* and *C. crescentus*, MreB forms axial helices which are dynamically rearranged during the cell cycle [59]. Also, MbI, a MreB paralog in *Bacillus subtilis*, is involved in organization of a axial spiral [60]. It is also shown that MreB locates under the inner surface of the cytoplasmic membrane [61], and directs lateral cell wall synthesis in a helical pattern [52,62]. The previous findings and our data support a model where the TorR protein targets the old cell poles by interacting with the C-terminal domain of MreB with the assistance of energy from ATP and the DnaK chaperone. It is reasonable to consider that the C-terminus of MreB may be exposed to the outside of the MreB helical filament, providing an interacting scaffold for TorR delivery or docking. The TorR delivery by MreB and DnaK chaperone is propelled by the energy from ATP; indeed, the absence of ATP leads to defects in the delivery of TorR to the old cell poles. In agreement with the model, the MreB helical structure requires ATP to bind to its nucleotide-binding pocket [63] and DnaK uses the free energy of ATP and hydrolysis to regulate their affinity for protein substrate [64]. Thus, it may be suggested that ATP-bound DnaK interacts with TorR in high affinity and subsequently delivers TorR to function at the old cell poles. It should be noted that the TorS protein might play an important role in the delivery of TorR to the cell poles as a signaling partner [32]. We, however, cannot exclude contributions of the outer membrane proteins (OmpA and OmpT) and a transmembrane protein (MalF), a member of the phosphate-specific transport system (PstB), in the polar localization of TorR. These proteins are pulled down with TorR-GFP in immunoprecipitation. Also, MalF and PstB are found at one pole as a focus and OmpT at two poles as two foci while OmpA seems to be arranged in a helical structure (Figure S8) although depletion of the proteins does not change the polar localization of TorR.

Our data also suggest that FtsZ-ring formation seems to be a signal for assembly of a new TorR focus in a newborn cell since the defect in formation of the FtsZ-ring by inactivation of FtsZ84 results in enlargement of the old TorR-GFP focus without assembling a new ‘normal’ focus (Figure 6). It is likely that the number of new FtsZ-rings and of new TorR foci could be the same because multi-TorR-GFP foci form in ∆*minCDE* cells (Figure 6) where multi FtsZ-rings also form in the elongated cells [56]. However, the question of how TorR identifies the old cell poles remains elusive. A diffusion and capture model suggests that a protein is diffused in the cytoplasm and is trapped at the poles transiently by interacting with a polar protein that is already localized at the cell poles [65]. It is also suggested that high-order protein assemblies are favored in membrane regions of stronger curvature [66] and energetically outside of the nucleoid region [67]. Composition of the cytoplasmic membrane and peptidoglycan differs between cell poles and the rest of the cell envelope, the differences serving as cues for the polar localization of proteins [68]. It could also be possible that the MreB helical filament has polarity, of which the C-terminal end extends to the old poles, providing a docking site for TorR protein to form a focus, while the N-terminal end occupies the new poles. The possible polarity of MreB helical filament may mark the old cell poles. Indeed, the *C. crescentus* TipN protein marks the site of the most recent division by identifying the new pole. The positional information can be used to establish and maintain the orientation of the polarity axis, which is crucial for polar morphogenesis and division [69].

We show that TorR-GFP is soluble whereas inclusion bodies of OstA-GFP are insoluble after cell lysis (Figure 1c–f). IbpA-mCherry recognizes and binds to misfolded proteins or inclusion bodies [33,70], but does not overlap with TorR-GFP (Figure S1a,b). Thus, the TorR-GFP foci at the old cell poles are not inclusion bodies or misfolded protein aggregates. It is shown that misfolded protein aggregates positioning at the cell poles is neither dependent on MreB nor on DnaK, DnaJ and ClpB [34]. We show that the formation, polar localization and regulatory effect of TorR-GFP focus on gene expression depend on its interaction with MreB and DnaK with energy provided by ATP (Figure 4, Figure 5 and Figure 7). These findings suggest that TorR-GFP focus at the old cell poles is a functional high-order protein structure.

### 4.2. A Spatial Control for Correct Timing of Gene Expression during the Cell Cycle

Timing of gene expression during the cell cycle, of some key genes specifically, is crucial for progression of the cell cycle and is a universal process. About 400–800 genes in eukaryotic cells including *Arabidopsis thaliana*, *Homo sapiens*, *Saccharomyces cerevisiae*, *Schizosaccharomyces pombe* [71] as well as bacteria [4] and 160 genes in archaeon [72] are expressed in a cyclic manner. Several molecular processes have been shown to regulate expression of key regulator genes in a cyclic manner: (i) a dynamic interaction of activator and repressor proteins with promoters (the dynamic cell cycle-dependent interaction of the *CtrA* suppressor and the *GcrA* activator with the *ctrA* promoter in *C. crescentus*) [6,73]; (ii) accumulation and/or degradation of key regulators (the cell cycle-dependent proteolysis of CtrA and DnaA in *C. crescentus*) [74]; and (iii) a dynamic change in protein activity (hydrolysis of ATP-DnaA or reactivation of ADP-DnaA, phosphorylation or dephosphorylation of CtrA) [12,75]. Here, we present a different control mechanism for correct timing of gene expression during the cell cycle, in which the polar localized TorR protein interacts with its target genes after the end of replication (Figure 2), and, in turn, the interaction represses or activates a number of genes in a cell cycle-dependent manner. The control disappears as soon as the interaction of TorR with its target genes ends (Figure 2 and Figure 3). Deletion of either the SR or the RR of the TorR protein or the absence of ATP abolishes its polar localization, interaction with MreB or DnaK and regulatory function for gene expression (Figure 7). The observations allow us to conclude that spatial control for correct timing of gene expression in the cell cycle is highly regulated.

Distribution patterns of the response regulators of the two-component signal transduction systems in the *E. coli* cells are different [20]. Several of them localize at one pole of the cells as a focus (RstA-GFP, CheB-GFP, CheY-GFP and YpdB) [20]. OmpR-GFP (Figure S8) fills two ends of the cell, leaving a space for the nucleoid in the middle, and many response regulators are diffused over the cytoplasma (BaeR-GFP, EvgA-GFP, NarL-GFP, PhoP-GFP) [20]. Very interestingly, CitB-GFP forms several foci along the long axis in some cells, or appears only in the middle of the cell in other cells [20]. We believe that the specific distribution pattern of a response regulator might be tightly associated with its function as a transcription factor. It is most likely that the response regulators localize in a limited space (at poles or middle of cells) to interact with their target genes only in a specific period of the cell cycle. Thus, they regulate expression of some genes in a cell-cycle-dependent manner. In contrast, the others, are which are diffused over the cytoplasm, control gene expression by phosphorylation or dephosphorylation as a switch. In line with this proposal, phosphorylated as well as unphosphorylated TorR bind to *tor* boxes in vivo to regulate expression of the *tor* operon [18], and this may apply to the functioning of the spatial control mechanism.

## Supplementary Materials

All supplementary data are available online at www.mdpi.com/2073-4425/8/1/01/s1, Movie S1: Subcellular localization of TorR-GFP in live cells. Figure S1: TorR polar focus is distinct from inclusion bodies, Figure S2: Polar TorR-GFP remains soluble upon temperature-shifts, Figure S3: The wild-type TorR regulates expression of genes in a cell-cycle-dependent manner, Figure S4: The SDS-PAGE gel analysis of proteins bound to GFP antibody on GFP-Trap beads, Figure S5: Subcellular localizations of the TorR-GFP in 6 mutants, Figure S6: MreB is required for localization of TorR at the old pole of cell, Figure S7: Protein-protein interactions were detected by a bacterial two-hybrid system, Figure S8: Subcellular localizations of the proteins pulled-down with TorR-GFP, Figure S9: The truncated TorR-GFP protein is expressed at the same level as the wild-type TorR-GFP, Table S1: Expression of genes at the 45 min time point relative to that at 0 min time after release from initiation-arrest in either presence or absence of TorR, Table S2: Proteins pulled-down with TorR-GFP, Table S3: Bacterial strains, Table S4: Plasmids, Table S5: Primers used.
