# Supplementary material for: A Spatial Control for Correct Timing of Gene Expression during the Escherichia coli Cell Cycle
Source: Genes (Basel). 2016 Dec 23;8(1):1. doi: 10.3390/genes8010001 (PMC5294996; doi:10.3390/genes8010001)
Supplement: Supplementary file 1 [file genes-08-00001-s001.zip › genes-149818-sup-final/genes-149818-supplementary-final.docx]

Supplementary Materials: A Spatial Control for Correct Timing of Gene Expression during the Escherichia coli Cell Cycle

**Yuan Yao, Lifei Fan, Yixin Shi, Ingvild Odsbu and Morigen**


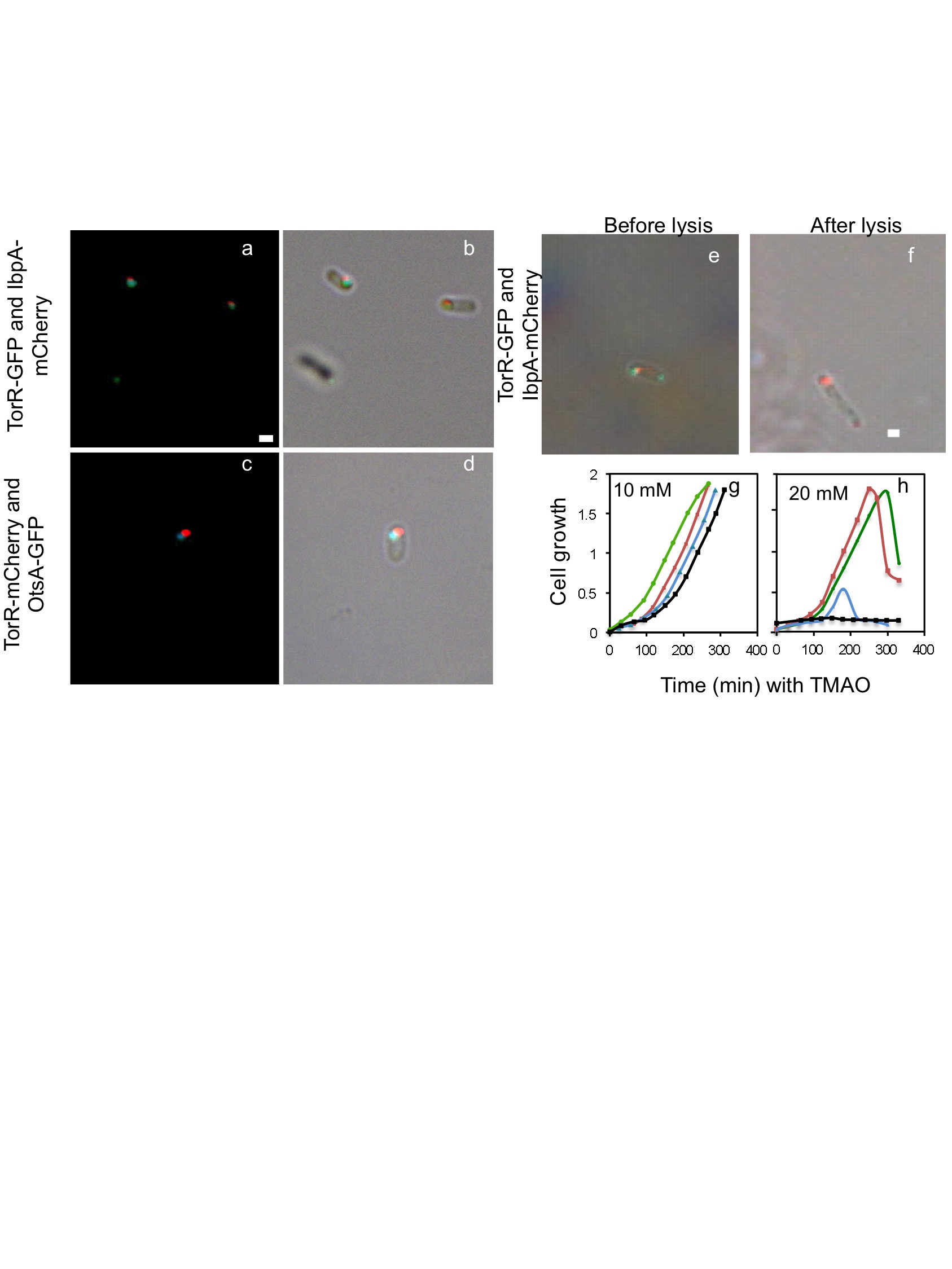


**Figure S1.** TorR polar focus is distinct from inclusion bodies. (**a**–**d**) W3110∆*torR* cells containing the pTorR-GFP and pIbpA-mCherry plasmids (**a**,**b**) or the Δ*torR* cells carrying the pTorR-mCherry and pOtsA-GFP plasmids (**c**,**d**) were exponentially grown in ABTGcasa medium (see Material and Methods in detail) with IPTG to induce expression of the protein fusions at 37 °C. The TorR-GFP and IbpA-mCherry or TorR-mCherry and OtsA-GFP protein fusions were then visualized by fluorescence microscopy. (**a)** and (**b)** indicate cells with TorR-GFP and IbpA-mCherry fusions. (**c**,**d**) stand for cells with TorR-mCherry and OtsA-GFP fusions. The scale bars represent 1 μm; (**e**,**f**) Cells expressing TorR-GFP and IbpA-mCherry (**e**,**f**) were visualized in 1.2% agarose pads of ABTGcasa medium before and after cell lysis (2 mg/mL lysozyme, 0.1% Triton X-100, and 10 mM EDTA). The scale bars represent 1 μm; (**g**,**h**) Exponentially growing wild-type W3110 (green), W3110Δ*torR* (blue), W3110Δ*torR*/pACYC177-*torR* (red) and W3110Δ*torR*/pACYC177 (black) cells were diluted 10 times in ABTGcasa with 10 mM (**g**) or 20 mM TMAO (**h**), and growth curves were monitored. The X axis presents time with TMAO and the Y axis indicates cell density (OD_450_). The values are the average of three experiments.


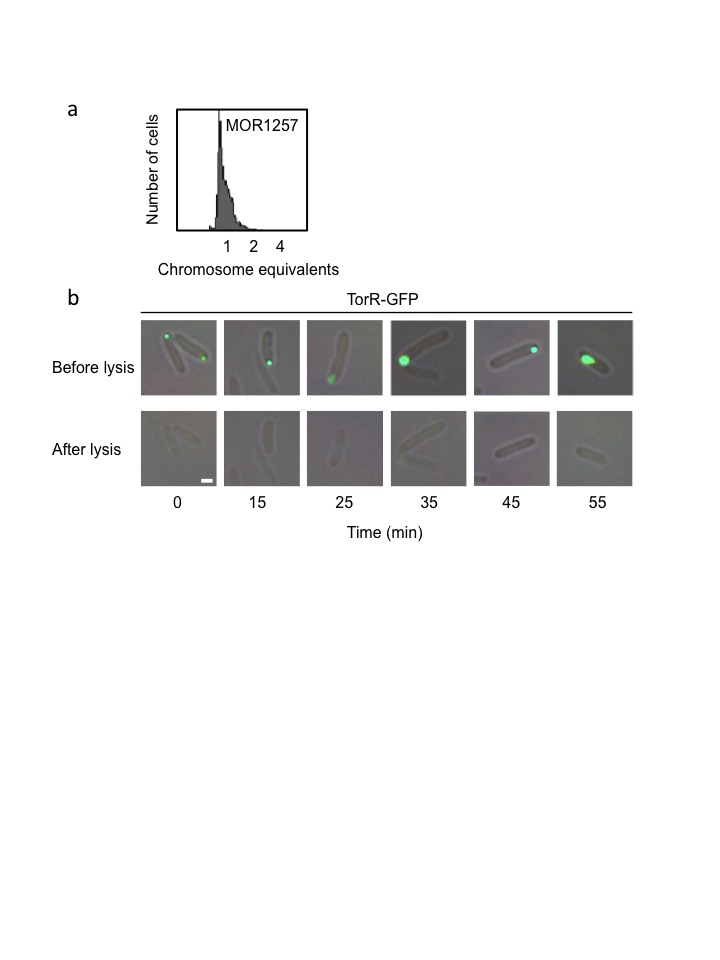


**Figure S2**. Polar TorR-GFP remains soluble upon temperature-shifts. (**a**) Exponentially growing W3110Δ*torRdnaC2* cells carrying pTorR-GFP (MOR1257) in ABTGcasa medium with IPTG for expression of TorR-GFP were shifted from the permissive temperature (30 °C) up to the non-permissive temperature (42 °C). After 2 h of incubation at 42 °C, the synchronized cells were shifted down to 30 °C, sampled at the 0 min point, and fixed in 70% ethanol and then stained with Hoechst33258. The chromosome equivalents per cell were measured by flow cytometer. The X-axis indicates chromosome equivalents per cell, and the Y-axis represents number of cells measured;
(**b**) The synchronized W3110Δ*torRdnaC2* cells carrying pTorR-GFP were taken at the time intervals after synchronization as indicated. Cells expressing TorR-GFP were visualized in 1.2% agarose pads of ABTGcasa medium before (upper panels) and after (lower panels) cell lysis (2 mg/mL lysozyme, 0.1% Triton X-100, and 10 mM EDTA). The scale bars represent 1 μm.


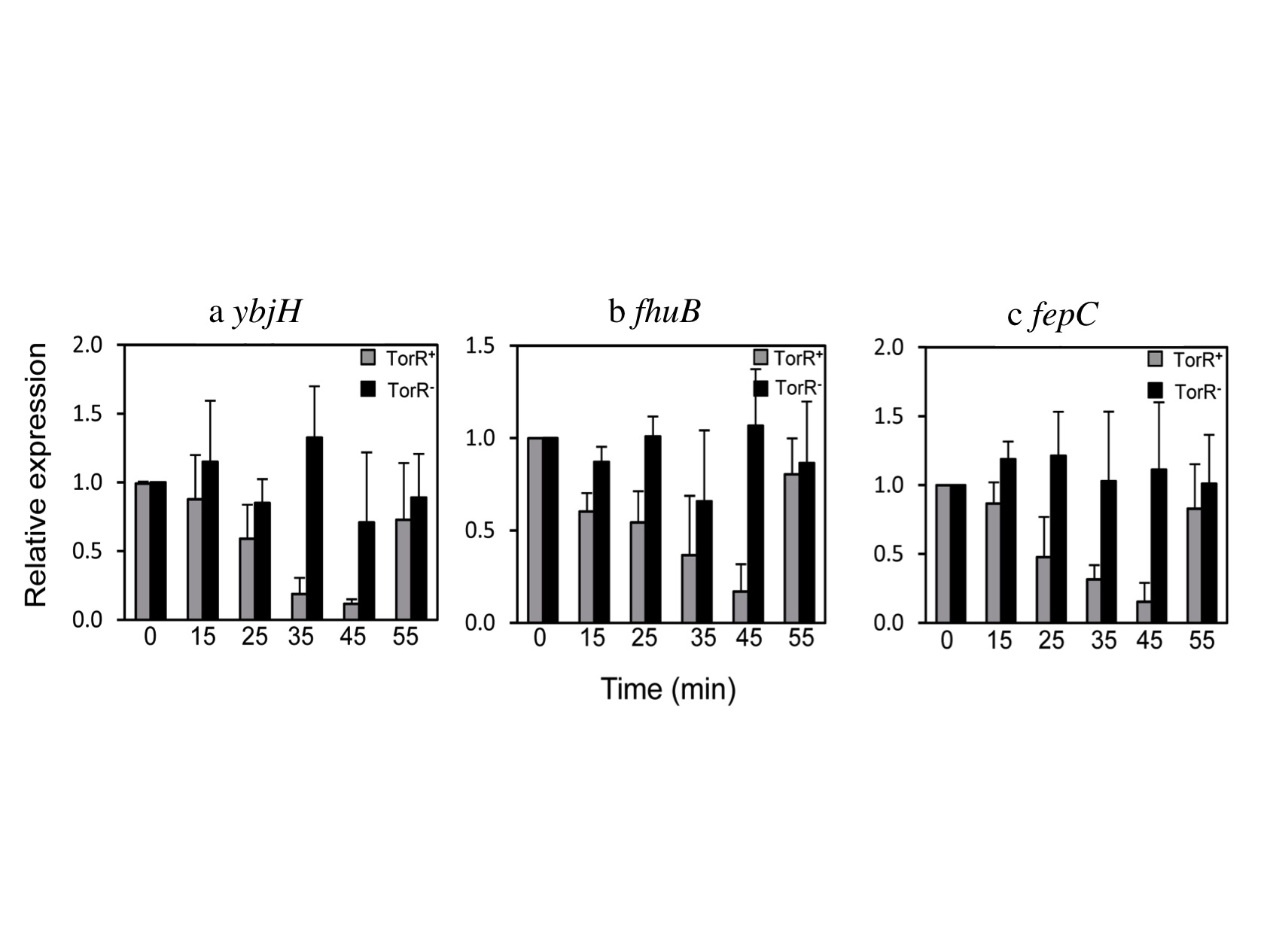


**Figure S3.** The wild-type TorR regulates expression of genes in a cell-cycle-dependent manner. Synchronization and preparation of W3110*dnaC2* and W3110Δ*torRdnaC2* cells were as described in the legend to Figure 3. Total RNA isolation, cDNA synthesis and performance of RT-qPCR were as mentioned in Materials and Methods and Figure 3. The X-axis indicates the time intervals after shift-down of the culture to the permissive temperature (30 °C), the Y-axis represents the relative expression of each gene. The grey bars are for expressions of genes in the cells with TorR and the black bars are for that in the cells without TorR. The values are the average of three experiments. The error bars are as indicated. All the genes analyzed are as indicated (**a**–**c**).


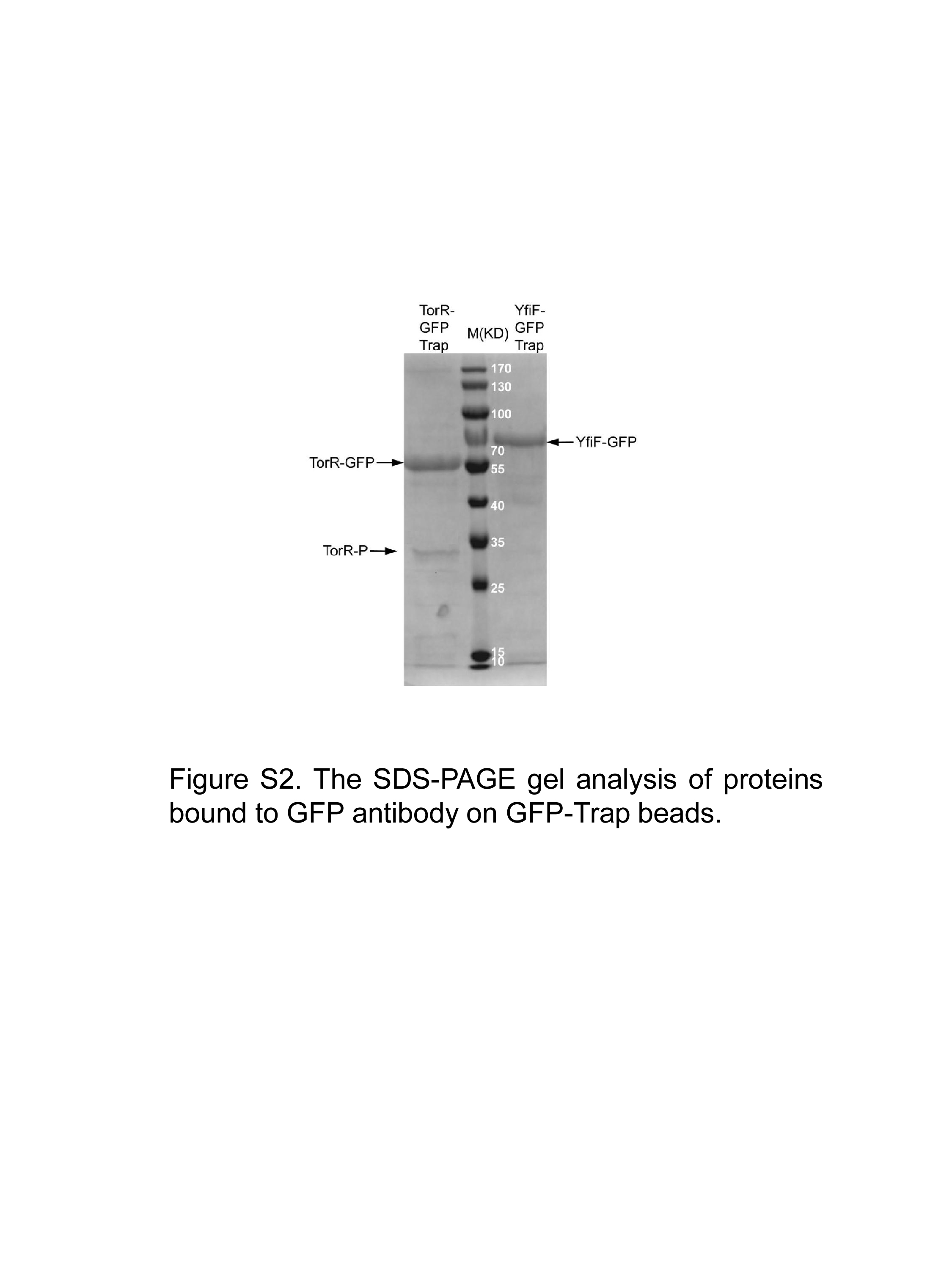


**Figure S4.** The sodium dodecyl sulfate polyacrylamide gel electrophoresis (SDS-PAGE) analysis of proteins bound to GFP antibody on GFP-Trap beads. W3110Δ*torR* cells harboring pTorR-GFP were exponentially grown in ABTGcasa with IPTG for expression of TorR-GFP fusion at 37 °C. The experiment also included W3110 cells carrying pYfiF-GFP as a control. The cell lysate containing TorR-GFP or YfiF-GFP was loaded onto GFP-Trap beads, respectively. Proteins bound to beads were eluted by elution buffer and analyzed by SDS-PAGE gel. The proteins were stained in Coommasie Blue and the band labeled by TorR-P was cut for mass-spectrometry. The molecular weight of TorR-GFP is 54 kD. TorR-GFP, TorR-P and markers are as indicated.


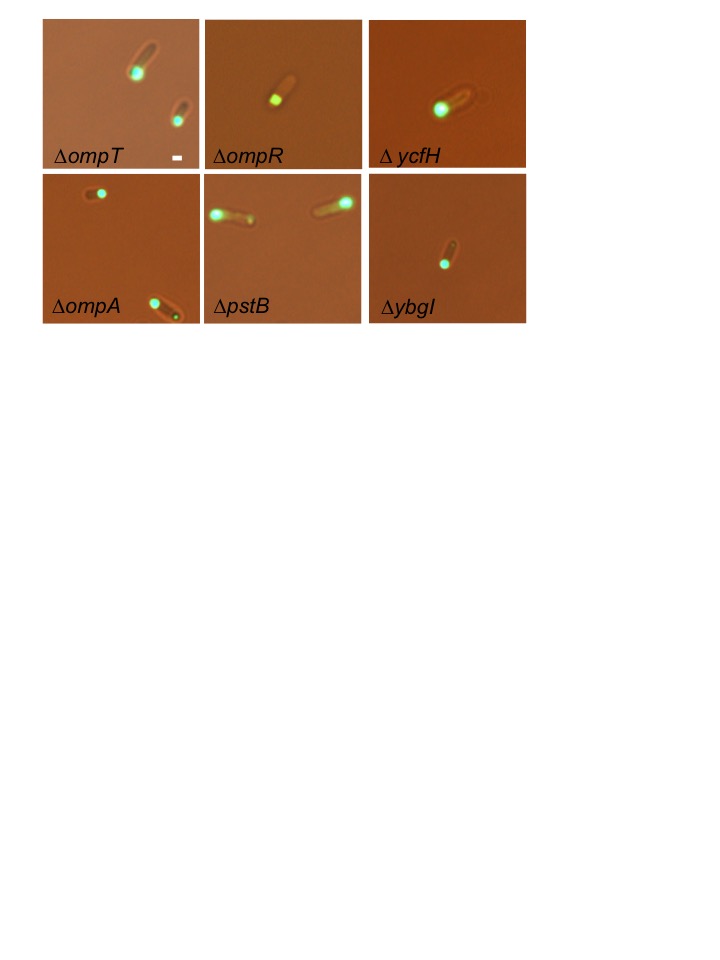


**Figure S5.** Subcellular localization of the TorR-GFP in six mutants. Exponentially growing cells harboring pTorR-GFP in ABTGcasa with IPTG for expression of the TorR-GFP fusion at 37 °C were fixed in 70% ethanol. Subcellular localizations of the TorR-GFP fusion was visualized as described in the legend of Figure 1. The scale bar represents 1 μm.


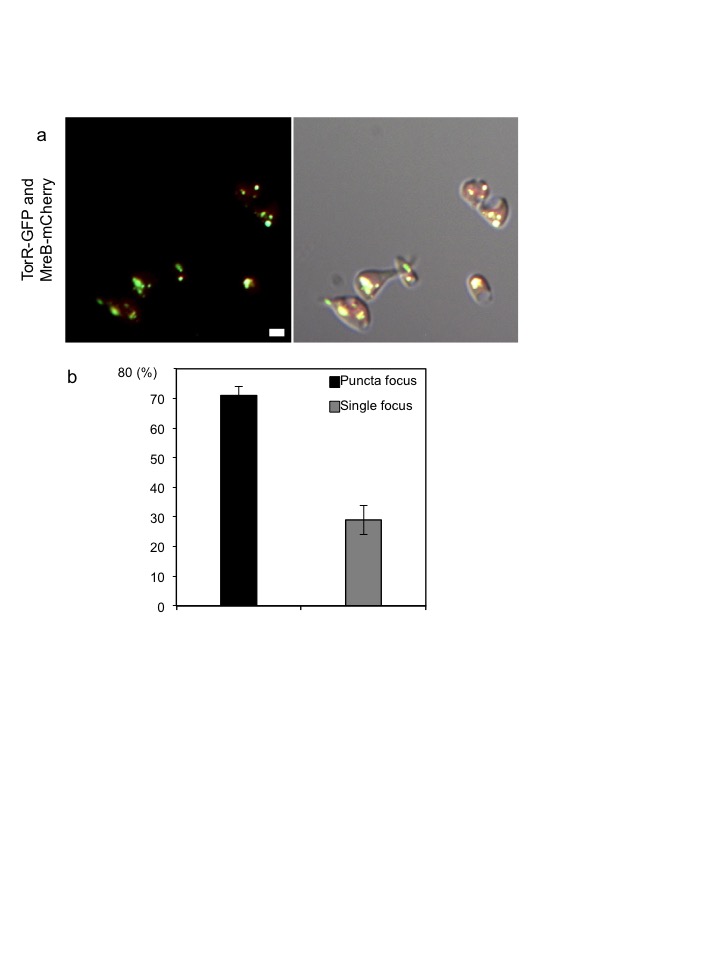


**Figure S6.** MreB is required for localization of TorR at the old pole of cell. (**a**) W3110Δ*torR* cells containing the pTorR-GFP and pMreB-mCherry plasmids were exponentially grown in ABTGcasa medium with IPTG to induce expression of the protein fusions at 37 °C. The TorR-GFP and MreB-mCherry protein fusions were then visualized by fluorescence microscopy. The scale bars represent 1 μm; (**b**) Exponentially growing W3110Δ*torR*/pTorR-GFP cells in ABTGcasa with IPTG at 37 °C were treated with A22 (10 μg/mL) for 6 h to inhibit MreB, and then fixed in 70% ethanol. The subcellular localization of TorR-GFP was visualized by fluorescence microscopy. The percentage of cells with a single focus or multi punctae of TorR-GFP was calculated. About 100 cells were included in each calculation. The values are the average of three individual experiments and standard errors are given as the error bars.


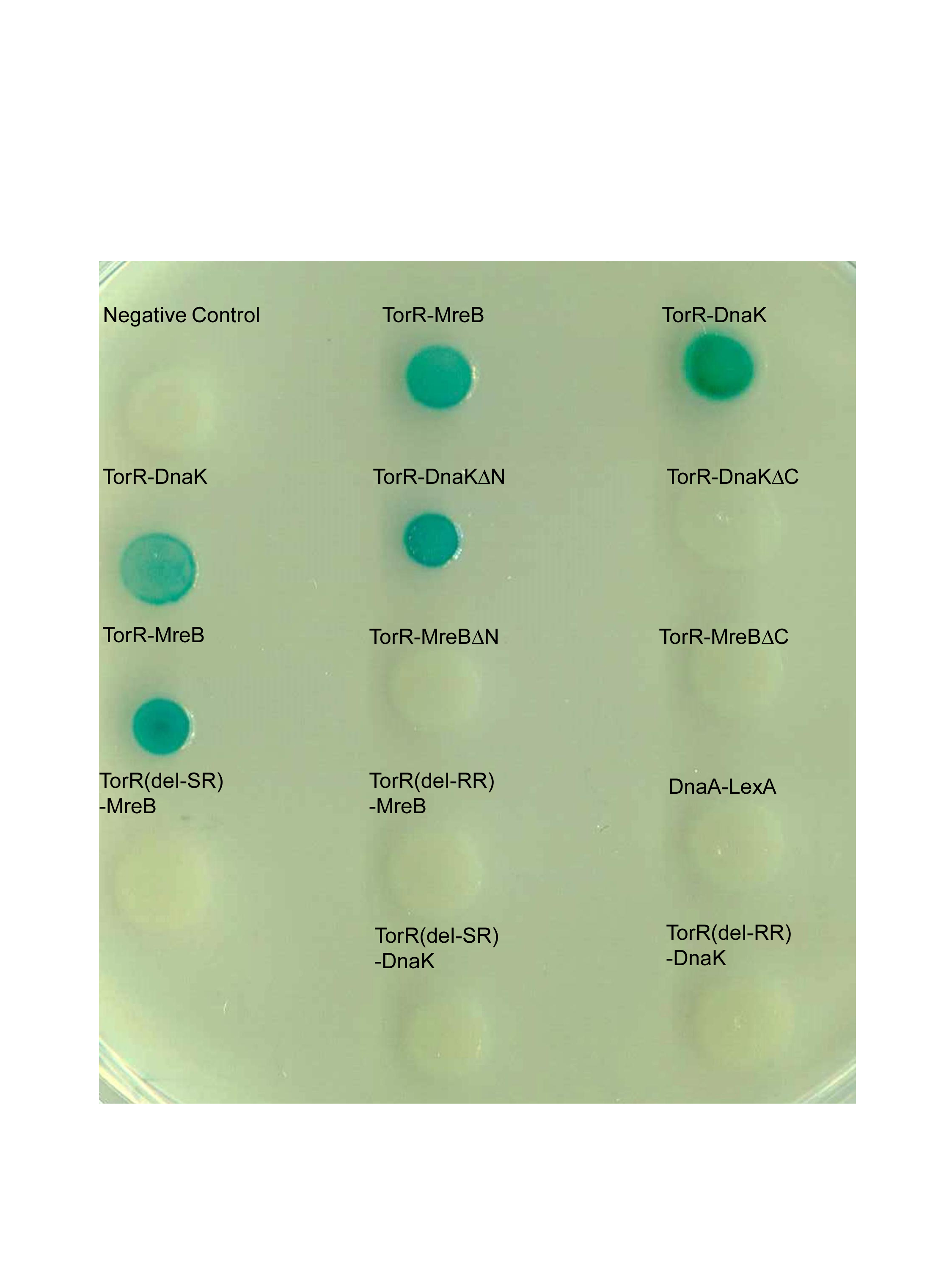


**Figure S7.** Protein–protein interactions were detected by a bacterial two-hybrid system. In the bacterial two-hybrid system, when two proteins interact the *lacZ* gene is expressed in a cAMP/CRP (cAMP receptor protein)-dependent mode, forming blue colonies on LB plates containing X-gal and IPTG whereas two proteins detected do not interact, resulting in white colonies. BTH101 cells were co-transformed with plasmid pairs encoding T18 and T25, respectively, or T18-DnaA and T25-LexA fusions as negative controls, then plated on the LB plates mentioned above with the required antibiotics, incubated at 30 °C for 30 h. Other plasmid pairs were used in the detection as described in the legends for Figures 4 and 7. To exclude “false” blue colonies, 3 μL of the bacterial culture from each transformant after grown in LB for 2 h was mounted the same plate as described above, incubated at 30 °C for 30 h. All transformants illustrating different protein-protein interactions were simultaneously tested on one plate to have the same reaction condition. The blue bacterial halos indicate protein-protein interactions while the white halos show no interaction. Protein pairs under detection are as indicated.


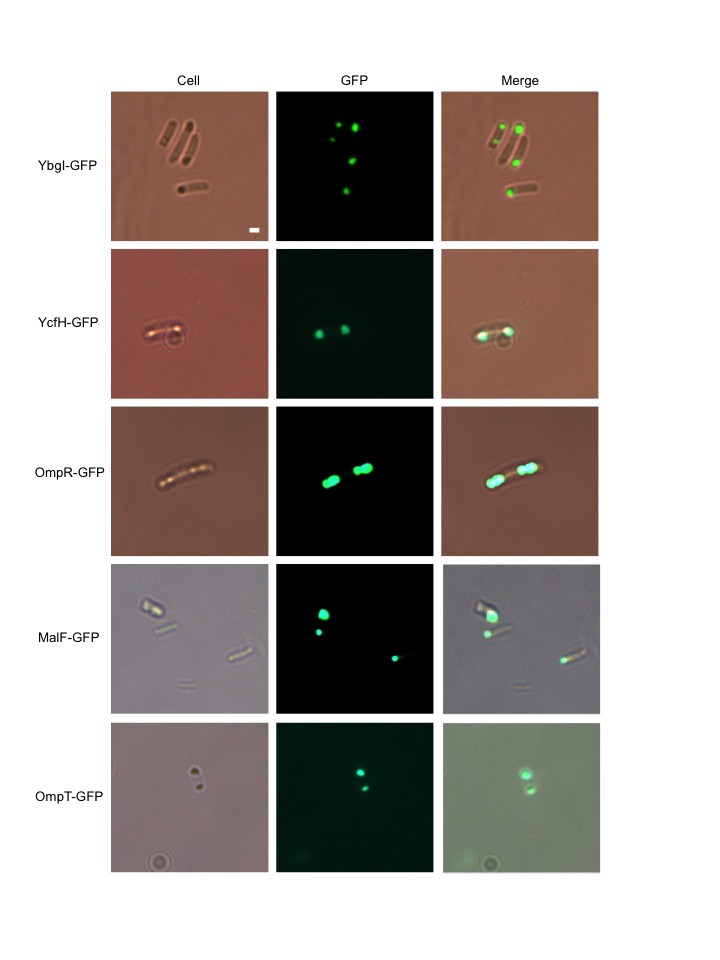


**
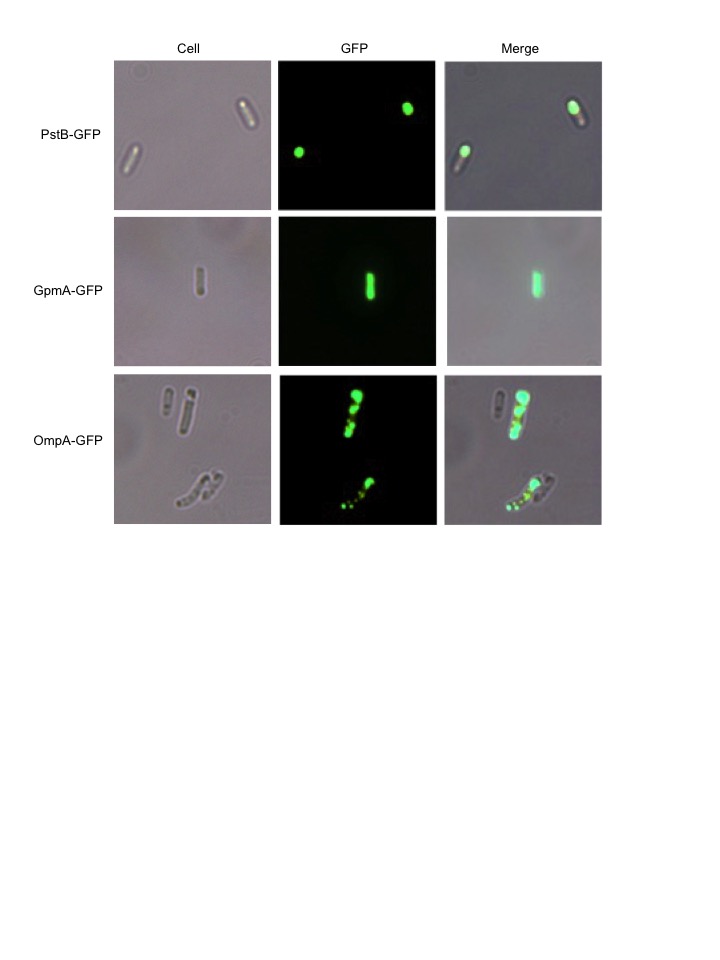
**

**Figure S8.** Subcellular localizations of the proteins pulled-down with TorR-GFP. Exponentially growing cells harboring plasmids containing the gene fused to *gfp* in ABTGcasa with IPTG for expression of the GFP fusions at 37 °C were fixed in 70% ethanol. The protein fusions are as indicated on the left. Subcellular localizations of the fusion proteins were visualized as described in the legend to Figure 1. The scale bar represents 1 μm.


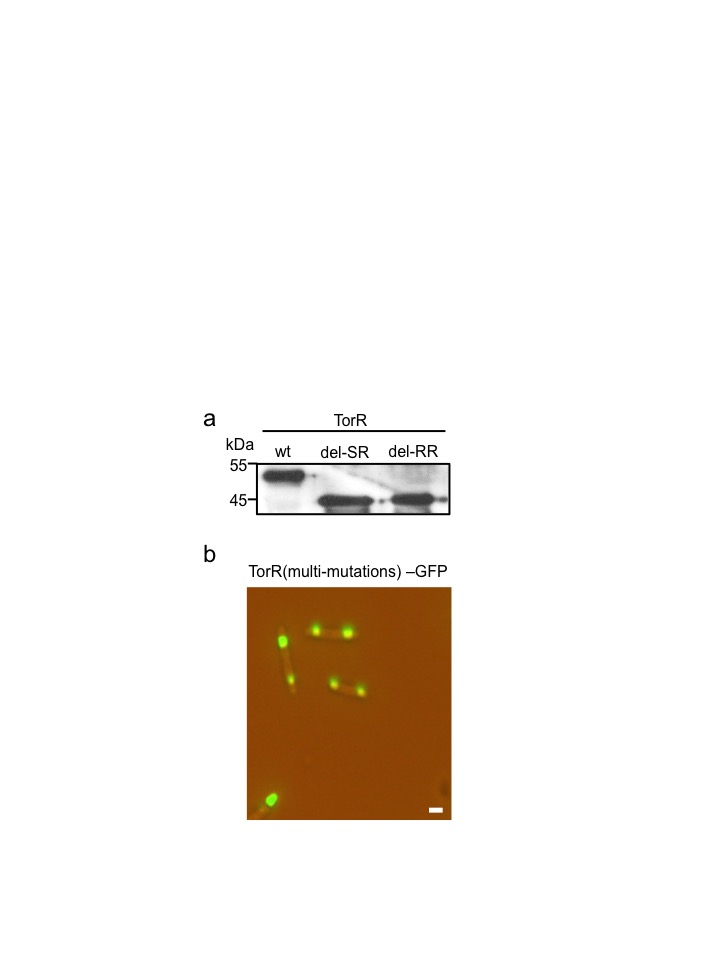


**Figure S9.** The truncated TorR-GFP is expressed to the level of the wild-type TorR-GFP. (**a**) W3110Δ*torR* cells harbouring pTorR-GFP, pTorR(del-SR)-GFP or pTorR(del-RR)-GFP were exponentially grown in ABTGcasa with IPTG for expression of TorR-GFP fusion at 37 °C and harvested by centrifugation at 4 °C. Total protein amount in a fixed volume of cell extract was determined by a colorimetric assay (BCA kit). Proteins were separated by SDS-PAGE gel and transferred to a polywinylidene difluoride (PVDF) membrane through semi-dry blotting. The membrane was probed with anti-mouse GFP antibody. The molecular weights are as indicated; (**b**) Exponentially growing cells in ABTGcasa with IPTG for expression of the
TorR(multi-mutations)-GFP fusions at 37 °C were fixed in 70% ethanol. Subcellular localization of the fusion protein was visualized as described in the legend to Figure 1. The scale bar represents 1 μm.

**Table S1.** Expression of genes at the 45 min time point relative to that at 0 min time after release from initiation-arrest in either presence or absence of TorR.

| **Gene** | **Gene ID** | **Changes in Expression** | |
| --- | --- | --- | --- |
|  |  | **TorR^+^** | **TorR^−^** |
| *fecA* | 12932560 | 0.3032 | NC |
| *ymgB* | 12934111 | 0.3347 | NC |
| *nrdE* | 12930227 | 0.2502 | NC |
| *entF* | 12930901 | 0.2131 | NC |
| *ybjJ* | 12933837 | 0.3123 | NC |
| *fecR* | 12932566 | 0.3048 | NC |
| *sufE* | 12931284 | 0.2095 | NC |
| *sufB* | 12931288 | 0.1914 | NC |
| *ybdB* | 12931988 | 0.276 | NC |
| *nrdI* | 12933274 | 0.2147 | NC |
| *entE* | 12930911 | 0.2246 | NC |
| *sufS* | 12931287 | 0.1793 | NC |
| *ymgC* | 12932749 | 0.3035 | NC |
| *sufC* | 12931285 | 0.1797 | NC |
| *ybjI* | 12933836 | 0.3088 | NC |
| *entA* | 12931990 | 0.2842 | NC |
| *entD* | 12931715 | 0.157 | NC |
| *fes* | 12930902 | 0.2544 | NC |
| *fhuB* | 12930741 | 0.2932 | NC |
| *nrdH* | 12931699 | 0.2459 | NC |
| *sufA* | 12931289 | 0.1968 | NC |
| *fepA* | 12934080 | 0.247 | NC |
| *sufD* | 12931286 | 0.1788 | NC |
| *entB* | 12931911 | 0.2554 | NC |
| *fhuE* | 12931948 | 0.1766 | NC |
| *entC* | 12931992 | 0.2958 | NC |
| *fepC* | 12930905 | 0.3721 | NC |
| *ybjH* | 12933835 | 0.4398 | NC |
| *bglJ* | 12933789 | 0.3395 | NC |
| *ftn* | 12934013 | 6.1772 | NC |

Cell synchronization and global transcription microarray analysis were as described in Materials and methods and the text. The relative expressions of genes were calculated by finding ratio of the values at 45 min time to that 0 min after release from initiation-arrest. The values < 0.5 indicate down-regulated expressions. The value > 2 shows up-regulated expression. ‘NC’ represents ‘No Change’ in expressions of genes at the 45 min relative to the 0 min. The gene ID indicates the Entry number of NCBI.

**Table S2.** Proteins pulled-down with TorR-GFP.

| **Accession No.** | **Protein Name** | **Protein Score** | **Unique Peptides Detected** | **Sequence Coverage (%)** |
| --- | --- | --- | --- | --- |
| 388479924 | RpsC | 110 | 10 | 54 |
| 388479921 | RplB | 29 | 7 | 33 |
| 388479833 | OmpR | 51 | 9 | 40 |
| 388479512 | PstB | 87 | 11 | 36 |
| 388479505 | AtpD | 27 | 6 | 17 |
| 388479285 | RplA | 229 | 16 | 56 |
| 388479242 | MreB | 21 | 5 | 20 |
| 388479188 | MlaF | 98 | 7 | 36 |
| 388478600 | PdxJ | 32 | 4 | 24 |
| 388477905 | ProQ | 31 | 5 | 28 |
| 388477349 | RluB | 22 | 5 | 18 |
| 388477180 | YcfH | 40 | 6 | 25 |
| 388477041 | OmpA | 92 | 9 | 30 |
| 388476851 | YbhA | 33 | 5 | 19 |
| 388476840 | GpmA | 54 | 6 | 23 |
| 388476812 | GltA | 72 | 6 | 17 |
| 388476802 | YbgI | 37 | 4 | 24 |
| 388476666 | OmpT | 36 | 9 | 30 |
| 388476316 | MetQ | 50 | 7 | 41 |
| 388476287 | RpsB | 213 | 13 | 50 |
| 388476136 | DnaK | 7 | 2 | 5 |

Immunoprecipitation by green fluorescent protein (GFP)-Trap and subsequently mass spectrometry analysis were as described in Materials and methods. ‘Accession No.’ represents the Entry number of NCBI or Uniprot. ‘The protein score’ indicates the value obtained by MS analysis. ‘Unique peptides detected’ represents the detected frequency of specific polypeptides belonging to a protein. ‘Sequence Coverage’ represents percentage of amino acids detected in total number of amino acids from a specific protein.

**Table S3.** Bacterial strains.

| **Strain** | **Genotype** | **Reference or Source** |
| --- | --- | --- |
| W3110 | Wild type F-lambda-IN(*rrnD-rrnE*)1 *rph*-*1* | [[76](#_ENREF_75)] |
| BW25113 | Wild type *rrnB3* ∆*lacZ4787 hsdR514* ∆(*araBAD*)*567*∆(*rhaBAD*)*568 rph-1* | [77] |
| BTH101 | F *cya-99 araD139 galE15 galK16 rpsL1* (Str^r^) *hsdR2 mcrA1 mcrB1* | [[23](#_ENREF_23)] |
| MOR1255 | W3110 *torR*::*kan* | This work |
| MOR166 | W3110 *dnaC2*(*Ts*)::*tet* | [27] |
| MOR1256 | W3110 *dnaC2*(*Ts*)::*tet*, *torR*::*kan* | This work |
| MOR314 | BW25113 *torR*::*kan* | [77] |
| MOR1226 | W3110 *ftsZ84*(*Ts*)::*tet*, *torR*::*kan* | This work |
| MOR1228 | MOR1226/pTorR-GFP | This work |
| MOR1230 | W3110 *torR*::*kan*/pIbpA-mCherry/pTorR-GFP | This work |
| MOR1231 | W3110 *minCDE*, *torR*::*kan* | This work |
| MOR1233 | MOR1231/pTorR-GFP | This work |
| MOR1234 | W3110/pYfiF-GFP | This work |
| MOR1235 | W3110/pOstA-GFP | This work |
| MOR1236 | W3110 *torR*::*kan*/pTorR-mCherry/pOstA-GFP | This work |
| MOR1257 | MOR1256/pTorR-GFP | This work |
| MOR1258 | MOR1255/pTorR(del-SR)-GFP | This work |
| MOR1259 | MOR1255/pTorR(del-RR)-GFP | This work |
| MOR1260 | MOR1255/pTorR(multi-mutations)-GFP | This work |
| MOR1261 | MOR1255/pACYC177-*torR* | This work |
| MOR1262 | MOR1255/pACYC177 | This work |
| MOR1264 | MOR1255/pTorR-GFP | This work |
| MOR1890 | W3110 *dnaK*::*kan*/pTorR-GFP | This work |
| MOR1891 | W3110 *ybgI*::*kan*/pTorR-GFP | This work |
| MOR1892 | W3110 *ompR*::*kan*/pTorR-GFP | This work |
| MOR1893 | W3110 *ompA*::*kan*/pTorR-GFP | This work |
| MOR1894 | W3110 *pstB*:: *kan*/pTorR-GFP | This work |
| MOR1897 | W3110 *ompT*::*kan*/pTorR-GFP | This work |
| MOR1899 | W3110 *ycfH*::*kan*/pTorR-GFP | This work |
| MOR1900 | W3110 *ybgI*::*kan*/pYbgI-GFP | This work |
| MOR1901 | W3110 *ompR*::*kan*/pOmpR-GFP | This work |
| MOR1902 | W3110 *gpmA*::*kan*/pGpmA-GFP | This work |
| MOR1903 | W3110 *pstB*:: *kan*/pPstB-GFP | This work |
| MOR1905 | W3110 *mlaF*::*kan*/pMlaF-GFP | This work |
| MOR1906 | W3110 *ompT*::*kan*/pOmpT-GFP | This work |
| MOR1907 | W3110 *ompA*::*kan*/pOmpA-GFP | This work |
| MOR1908 | W3110 *ycfH*::*kan*/pYcfH-GFP | This work |
| MOR2177 | MOR1256/pTorR(del-SR)-GFP | This work |
| MOR2178 | MOR1256/pTorR(del-RR)-GFP | This work |
| MOR2179 | MOR1256/pTorR(multi-mutations)-GFP | This work |
| MOR2274 | W3110 *torR*::*kan*/pMreB-mCherry/pTorR-GFP | This work |

**Table S4.** Plasmids.

| **Plasmid** | **Description** | **Reference or Source** |
| --- | --- | --- |
| pCA24N | rep _pMB1_Cm^R^*lacI^q^*p_pT5-_*_lac_*t*_his_*GFP | [[20](#_ENREF_20)] |
| pTorR-GFP | *torR* fused to GFP on pCA24N | [[20](#_ENREF_20)] |
| pTorR(del-SR)-GFP | pTorR-GFP with deletion of 6–74 aa in TorR | This work |
| pTorR(del-RR)-GFP | pTorR-GFP with deletion of 156–224 aa in TorR | This work |
| pTorR(multi-mutations)-GFP | pTorR-GFP with multiple site-directed mutations of S178N,R179H,T198N,L210P,G222A | This work |
| pGpmA-GFP | *gpmA* fused to GFP on pCA24N | [[20](#_ENREF_20)] |
| pOmpR-GFP | *ompR* fused to GFP on pCA24N | [[20](#_ENREF_20)] |
| pYbgI-GFP | *ybgI* fused to GFP on pCA24N | [[20](#_ENREF_20)] |
| pPstB-GFP | *pstB* fused to GFP on pCA24N | [[20](#_ENREF_20)] |
| pMlaF-GFP | *malF* fused to GFP on pCA24N | [[20](#_ENREF_20)] |
| pOmpA-GFP | *ompA* fused to GFP on pCA24N | [[20](#_ENREF_20)] |
| pYcfH-GFP | *ycfH* fused to GFP on pCA24N | [[20](#_ENREF_20)] |
| pOmpT-GFP | *ompT* fused to GFP on pCA24N | [[20](#_ENREF_20)] |
| pOtsA-GFP | *otsA* fused to GFP on pCA24N | [[20](#_ENREF_20)] |
| pYfiF-GFP | *yfiF* fused to GFP on pCA24N | [[20](#_ENREF_20)] |
| pACYC177 | rep_p15A_Ap^R^ (*bla*) Km^R^(*kan*) | [78] |
| pACYC177-*torR* | *torR* gene with its native promoter inserted into pACYC177 | This work |
| pcDNA3-*mCherry* | *mCherry* gene on pcDNA3 ( pcDNA3 derivative) | This work |
| pIbpA-mCherry | *ibpA* gene with its native promoter fused to *mCherry* and inserted into pACYC177 | This work |
| pTorR-mCherry | *torR* gene with its native promoter fused to *mCherry* and inserted into pACYC177 | This work |
| pMreB-mCherry | *mreB* gene with its native promoter fused to *mCherry* and inserted into pACYC177 | This work |
| pKNT25 | rep_p15A_Km^R^ p*_lac_T25*( pSU40 derivative) | [[22](#_ENREF_22)] |
| pUT18 | rep_ColE1_Ap^R^ p*_lac_T18*(pUC19 derivative) | [[22](#_ENREF_22)] |
| pKNT-*torR* | *torR* fused to T25 on pKNT25 | This work |
| pUT-*mreB* | *mreB* fused to T18 on pUT18 | This work |
| pUT-*dnaK* | *dnaK* fused to T18 on pUT18 | This work |
| pKNT-*torR*(del-SR) | pKNT-*torR* with deletion of 6–74 aa in TorR | This work |
| pKNT-*torR*(del-RR) | pKNT-*torR* with deletion of 156–224 aa in TorR | This work |
| pUT-*mreB*_∆2–90_ | pUT-*mreB* with deletion of 2–90 aa in MreB | This work |
| pUT-*mreB*_∆180–281_ | pUT-*mreB* with deletion of 180–281 aa in MreB | This work |
| pUT-*dnaK*_∆1-100_ | pUT-*dnaK* with deletion of 1–100 aa in DnaK | This work |
| pUT-*dnaK*_∆510-639_ | pUT-*dnaK* with deletion of 510–639 aa in DnaK | This work |
| pUT-*ftsZ* | *ftsZ* fused to T18 on pUT18 | This work |

**Table S5.** Primers used.

| **Name of Primer** | **Sequences (5' to 3')** | **Usage** |
| --- | --- | --- |
| dnaK-for | CCCAAGCTTGATGGGTAAAATAATTGGTAT(HindIII) | To fuse *dnaK* to T18  in pUT18 |
| dnaK-rev | CGGGATCCTCTTTTTTGTCTTTGACTTCTTC(BamHI) |  |
| DPR-for | CCATCGATGGGATTATTCTGGTTACC(ClaI) | To delete the SR domain of TorR in pTorR-GFP |
| DPR-rev | CCATCGATATAACAATGTGATGTGGC(ClaI) |  |
| DPD-for | CCATCGATCTTAGCCGCTGATGTGTGC(ClaI) | To delete the RR domain of TorR in pTorR-GFP |
| DPD-rev | CCATCGATAATCGGCTCGCCATCCCG(ClaI) |  |
| UPR-for | CCCTCGAGGGGATTATTCTGGTTACC(XhoI) | To delete the SR domain of TorR in pKNT-*torR* |
| UPR-rev | CCCTCGAGATAACAATGTGATGTGGC(XhoI) |  |
| UPD-for | CCCTCGAGCTTAGCCGCTGATGTGTGC(XhoI) | To delete the RR domain of TorR in pKNT-*torR* |
| UPD-rev | CCCTCGAGAATCGGCTCGCCATCCCG(XhoI) |  |
| fts-for | CCCAAGCTTGATGTTTGAACCAATGGAAC(HindIII) | To fuse *ftsZ* into T18  in pUT18 |
| fts-rev | CGGGATCCTCATCAGCTTGTTACGCAGG(BamHI) |  |
| ibpA-for | CCGCTCGAGAACGTTTCGTGCTGC (XhoI) | To clone *ibpA* gene with its promoter from chromosomal DNA |
| ibpA-rev | CCCAAGCTTTTGATTTCGATACGGCG(HindIII) |  |
| mix2-for | CCCAAGCTTGTGAGCAAGGGCGAGGAGG(HindIII) | To clone *mCherry* from pcDNA3-mCherry |
| mix2-rev | CGGGATCCTTACTTGTACAGCTCGTCCATGCCG(BamHI) |  |
| mreB-for | CCGCTCGAGCGCGCTGCGTCTCATG(XhoI) | To clone *mreB* gene with its promoter from chromosomal DNA |
| mreB-rev | CCCAAGCTTCTCTTCGCTGAACAGGTCG(HindIII) | To fuse *mreB* into T18 on pUT18 |
| mre-rev | CGGGATCCTCCTCTTCGCTGAACAGGT(BamHI) |  |
| tor-for | CCCAAGCTTGATGCCACATCACATTGTTAT(HindIII) | To fuse *torR* into T25 on pKNT25 |
| tor-rev | CGGGATCCTCGCACACATCAGCGGC(BamHI) |  |
| torR-for | CCGCTCGAGTTGCTCGCTTCCAGTTTG(XhoI) | To insert *torR* with its native promoter on pACYC177 |
| torR-rev | CGGGATCCTGTCAGCCCACCGATTTT(BamHI) |  |
| torR1-for | CCCAAGCTTGCGCCAGTACCGACCAACG(HindIII) | To clone *torR* gene with its promoter from chromosomal DNA |
| torR1-rev | CGGGGTACCGCACACATCAGCGGCTAAG(KpnI) |  |
| mix1-for | CGGGGTACCGTGAGCAAGGGCGAGGA (KpnI) | To clone *mCherry* from pcDNA3-mCherry |
| mix1-rev | CGGGATCCTTACTTGTACAGCTCGTCCATGC(BamHI) |  |
| rplO-for | ATTCGGCTTCACTTCTCGTAA | To detect *rplO* transcription by RT-qPCR (size of product is 101 bp) |
| rplO-rev | CTTTCAGCGTGTTCAGGTCTA |  |
| sufB-for | ACCCAGTTAGCCACCGATGAG | To detect *sufB* transcription by qRT-PCR (size of product is 121 bp) |
| sufB-rev | ATGCGCGATAGGCGTTTAGAC |  |
| fhuB-for | CGGCGTTGAGCGTTTATG | To detect *fhuB* transcription by qRT-PCR  (size of product is 120 bp) |
| fhuB-rev | AAGGCCAGCCCGAGATTG |  |
| fecA-for | GAACTTTGTTACCCGTGCCAT | To detect *fecA* transcription by qRT-PCR  (size of product is 113 bp) |
| fecA-rev | CCATCAGGTTGTGCGTCTCTT |  |
| ybjH-for | CGTCTATGTGGCTCCTGC | To detect *ybjH* transcription by qRT-PCR  (size of product is 109 bp) |
| ybjH-rev | ATGGTCGTCAATCTGGTGTT |  |
| fepC-for | GATGGCGAGCACATTCAA | To detect *fepC* transcription by qRT-PCR  (size of product is 182 bp) |
| fepC-rev | GCTTTCGTTACCGCTTCTT |  |
| ftn-for | CATATGCAGCGTCTGTTTGAT | To detect *ftn* transcription by qRT-PCR  (size of product is 191 bp) |
| ftn-rev | GTTGGGTAGTCCTGATTGGTC |  |
| entB-for | CCCAGCCGAAAGAGCAGA | To detect *entB* transcription by qRT-PCR  (size of product is 168 bp) |
| entB-rev | GCGGAGAACGATGAAACG |  |
| nrdH-for | CAGTGCCACGCCACCAAA | To detect *nrdH* transcription by qRT-PCR  (size of product is 162 bp) |
| nrdH-rev | CGGACGGAAACCAGACCA |  |
| M178-for | AATCCGGGCGAAATTCTCAACCGTGAACGT | To mutate Ser to Asn at 178 of TorR in pTorR-GFP |
| M178- rev | TTGAGAATTTCGCCCGGATT CGTCACAAA |  |
| M179-for | AATCCGGGCGAAATTCTCAGCCATGAACGTCTG | To mutate Arg to His at 179 of TorR in pTorR-GFP |
| M179-rev | TGGCTGAGAATTTCGCCCGGATTCGT CACAAA |  |
| M198-for | AACCCTGACCTGCGCAACGTCGATGTG | To mutate Thr to Asn at 198 of TorR in pTorR-GFP |
| M198-rev | TTGCGCAGGTCAGGGTTTTCCACCCGA |  |
| M210-for | GTCGTTTACGTCATAAACCCAGCGCGGAT | To mutate Leu to Pro at 210 of TorR in pTorR-GFP |
| M210-rev | GGTTTATGACGTAAACGACGAATTAACAC |  |
| M222-for | TGACGCAACATGGTGAAGCTTATTTCTTAG | To mutate Gly to Ala at 222 of TorR in pTorR-GFP |
| M222-rev | GCTTCACCATG TTGCGTCACCAGTAAATC |  |
| MDN-for | TGCTCTAGACAGCACTTCATCAAACAAGTGCAC(*Xba*I) | To delete the 2–90 aa of MreB in pUT-*mreB* |
| MDN-rev | TGCTCTAGACATCAAGCTTGGCGTAATCATGGTC(*Xba*I) |  |
| MDC-for | TGCTCTAGACTGGCTTCCGACATCTCCG(*Xba*I) | To delete the 180–281 aa of MreB in pUT-*mreB* |
| MDC-rev | TGCTCTAGAACCGTTCAAGGAGATAACAGC(*Xba*I) |  |
| DDN-for | TGCTCTAGATGGGTCGAAGTTAAAGGCCAGAAAATG(*Xba*I) | To delete the 1-100 aa of DnaK in pUT-*dnaK* |
| DDN-rev | TGCTCTAGACATCAAGCTTGGCGTAATCATGGTC(*Xba*I) |  |
| DDC-for | CGGGATCCCCGGGTACCGAGCTCGAATTCAG(*Bam*HI) | To delete the 510-639 aa of DnaK in pUT-*dnaK* |
| DDC-rev | CGGGATCCATCTTCGTTCAGACCAGAAGAAGCCTTG(*Bam*HI) |  |

Underlined sequences are restriction sites as indicated in the table.
